# The apocarotenoid metabolite zaxinone regulates growth and strigolactone biosynthesis in rice

**DOI:** 10.1038/s41467-019-08461-1

**Published:** 2019-02-18

**Authors:** Jian You Wang, Imran Haider, Muhammad Jamil, Valentina Fiorilli, Yoshimoto Saito, Jianing Mi, Lina Baz, Boubacar A. Kountche, Kun-Peng Jia, Xiujie Guo, Aparna Balakrishna, Valentine O. Ntui, Beate Reinke, Veronica Volpe, Takashi Gojobori, Ikram Blilou, Luisa Lanfranco, Paola Bonfante, Salim Al-Babili

**Affiliations:** 1 Division of Biological and Environmental Science and Engineering, the BioActives Lab, King Abdullah University of Science and Technology, Thuwal, 23955-6900 Saudi Arabia; 2 Department of Life Sciences and Systems Biology, University of Torino, Viale Mattioli 25, Torino, 10125 Italy; 3 Computational Bioscience Research Center, King Abdullah University of Science and Technology, Thuwal, 23955-6900 Saudi Arabia; 4 Cell Biology, Institute for Biology II, Albert-Ludwigs University of Freiburg, D-79104 Freiburg, Germany; 5 Division of Biological and Environmental Sciences and Engineering, King Abdullah University of Science and Technology, Thuwal, 23955-6900 Saudi Arabia

## Abstract

Carotenoid cleavage dioxygenases (CCDs) form hormones and signaling molecules. Here we show that a member of an overlooked plant CCD subfamily from rice, that we name Zaxinone Synthase (ZAS), can produce zaxinone, a novel apocarotenoid metabolite in vitro. Loss-of-function mutants (*zas*) contain less zaxinone, exhibit retarded growth and showed elevated levels of strigolactones (SLs), a hormone that determines plant architecture, mediates mycorrhization and facilitates infestation by root parasitic weeds, such as *Striga* spp. Application of zaxinone can rescue *zas* phenotypes, decrease SL content and release and promote root growth in wild-type seedlings. In conclusion, we show that zaxinone is a key regulator of rice development and biotic interactions and has potential for increasing crop growth and combating *Striga*, a severe threat to global food security.

## Abstract (web summary)

Strigolactone and abscisic acid are carotenoid-derived plant hormones. Here the authors describe the identification of zaxinone, a further apocarotenoid metabolite, which down-regulates strigolactone content and is required for normal growth and development in rice.

## Introduction

Plants rely on hormones and other chemical signals to coordinate growth and developmental processes, to adapt to environmental changes, and to communicate with surrounding organisms^1^. Many of these signals originate from secondary metabolic pathways, such as carotenoid biosynthesis^2^, that provides precursors for the phytohormones abscisic acid (ABA)^3^ and strigolactones (SLs)^4^. SLs regulate plant development, best-known for determining shoot and root architectures, in accordance to nutrients availability^5,6^. In addition, SLs are rhizospheric signals released by roots, particularly under phosphate (Pi) starvation, to facilitate the recruitment of arbuscular mycorrhizal (AM) fungi and establish the widespread beneficial AM symbiosis^7,8^. However, SLs are also perceived by seeds of root parasitic plants, such as *Striga* spp., as germination signal ensuring host availability required for the survival of these obligate parasites^9^. Infestation by *Striga* spp. is a severe problem for agriculture in warm and temperate zones, causing enormous yield losses^10^, and a major threat to global food security^11^.

Carotenoids are isoprenoid pigments characterized by an extended, conjugated double bond system. Plant carotenoids consist of a common C_40_-skeleton, but differ by the number of their conjugated double bonds, stereo-configuration, the presence of oxygen, and end-group structures^2^. SLs, ABA, and other carotenoid-derived compounds, such as the vision chromophore retinal, are the result of an oxidative cleavage of specific double bond(s) in defined carotenoid precursor(s)^12,13^. This conversion is a common metabolic process yielding apocarotenoids, generally catalyzed by carotenoid cleavage dioxygenases (CCDs) represented in all taxa^13,14^. However, reactive oxygen species (ROSs) can also trigger carotenoid cleavage, for example in the formation of the signaling molecule cyclocitral^15^. Apocarotenoids themselves are substrates of several CCDs^16^ and frequently modified by different enzymes, such as cytochrome P450 enzymes^17^. The Arabidopsis genome encodes nine CCDs, including five 9-*cis*-epoxycarotenoid cleavage dioxygenases (NCED2, 3, 5, 6, and 9) involved in ABA biosynthesis^18^. The further enzymes represent the four other plant CCD subfamilies, designated as CCD1, CCD4, CCD7, and CCD8^19^. CCD1 enzymes are likely scavengers of destructed carotenoids^20^, generating a plentitude of different products from a wide range of carotenoids and apocarotenoids^21–23^. CCD4 enzymes cleave all-*trans*-cyclic carotenoids^24^, determining carotenoid content in different tissues^25^, and forming apocarotenoid pigments in some fruits^12^. Additionally, the Arabidopsis CCD4 may generate a yet unidentified signal required for normal plastid and leaf development^12^. CCD7 is a SL biosynthetic enzyme cleaving 9-*cis*-β-carotene formed by the all-*trans*/9-*cis*-β-carotene isomerase DWARF27 into 9-*cis*-β-apo-10′-carotenal^26^. CCD8 converts this intermediate into carlactone, the central intermediate of SL biosynthesis^26,27^ and the substrate of cytochrome P450 enzymes (711 clade), such as the rice orobanchol oxidase, that form SLs^17,28^.

A survey of grass CCDs revealed a clade missing in Arabidopsis^29^. In our study, we analyzed the distribution of this subgroup in plant kingdom, investigated the enzymatic activity of a rice representative encoded by *LOC_Os09g15240* in vitro, and characterized a corresponding loss-of-function mutant. This enabled us to identify a carotenoid-derived, growth-regulating metabolite that is required for normal rice growth and development, as well as a bona fide, undescribed plant CCD subfamily common in most land plants.

## Results

### Characterization of Zaxinone Synthase(ZAS)

**Fig. 1:**
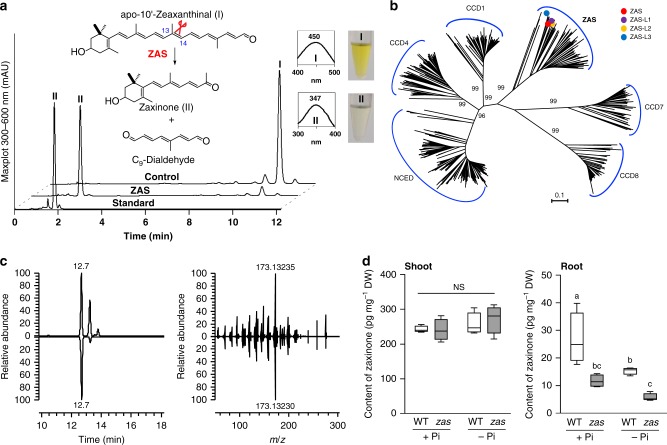
Characterization of ZAS. **a** HPLC analyses of in vitro incubation of ZAS with apo-10′-zeaxanthinal (I) yielded zaxinone (II) and a C_9_-dialdehyde. **b** Neighbour-joining tree of 782 plant CCD orthologues, showing bootstrap values on nodes of NCED, CCD1, CCD4, ZAS, CCD7, and CCD8 clusters only. See details of this tree and its bootstrap values in Supplementary Data 6. Circles represent sequences of ZAS (red) and its orthologues ZAS-L1 -L3. The scale bar indicates an estimated 0.1 change per amino acid. **c** Identification of endogenous zaxinone in rice, based on retention time (Left), accurate mass and MS/MS pattern (Right), in comparison to authentic standard. **d** Quantification of endogenous zaxinone in wild-type and *zas* mutant shoots (Left) and roots (Right) under normal (+Pi) and deficient (-Pi) phosphorus supply. Bars represent mean ± SD; *n* = 4 biological replicates. Statistical analysis was performed by one-way analysis of variance (ANOVA) and Tukey’s post hoc test. Different letters denote significant differences (*P* < 0.05), NS non-significant

First, we expressed the *LOC_Os09g15240* cDNA fused to *thioredoxin* in *Escherichia coli* cells and incubated the soluble fraction of these cells with different carotenoids and apocarotenoids in vitro (Supplementary Fig. 1). In this assay, we detected only the cleavage of 3-ΟΗ-apocarotenals (zeaxanthinals) with different chain lengths, i.e. apo-8′- (C_30_), apo-10′- (C_27_), and apo-12′-zeaxanthinal (C_25_), at the C13,C14 double bond, yielding apo-13-zeaxanthinone (3-OH-β-apo-13-carotenone, C_18_) identified in HPLC and LC-MS analysis by comparison to an authentic standard (Supplementary Fig. 1). We confirmed this reaction by incubating purified MBP-fusion of this enzyme with apo-10′-zeaxanthinal (Supplementary Fig. 2; Fig. 1a). For the sake of simplicity, we named this product zaxinone and the *LOC_Os09g15240*-encoded enzyme Zaxinone Synthase (ZAS). To further determine the substrate preference of ZAS, we measured the conversion of apo-10′- and apo-8′-zeaxanthinal over time, which revealed the former as the best substrate (Supplementary Fig. 3a). The cleavage of apo-10′-zeaxanthinal (C_27_) to zaxinone (C_18_) must yield a second product, presumably an instable dialdehyde with a C_9_ chain length. Derivatization with *O*-(2,3,4,5,6-pentafluorobenzyl)hydroxylamine hydrochloride enabled us to unequivocally identify this product, using UHPLC-MS/MS analysis (Fig. 1a; Supplementary Fig. 4). Next, we developed a protocol for extracting and detecting zaxinone from plant material, which allowed us to unambiguously demonstrate that this in vitro product is a natural metabolite present in rice (Fig. 1c) as well as in tobacco and *Arabidopsis* (Supplementary Fig. 5).

To explore the distribution of ZAS orthologues in plants, we extracted 748 sequences representing all CCD genes in 69 genomes of land plant species, including moss, fern, monocots, and dicots (Supplementary Data 1). A phylogenetic analysis suggested that *ZAS* represents the sixth CCD subfamily, besides the five currently known (Fig. 1b; Supplementary Data 6). *O. sativa* ZAS belongs to a cluster (Supplementary Data 7) that also includes three ZAS paralogues of *O. sativa*, ZAS-L1, ZAS-L2, and ZAS-L3 (Supplementary Data 7). ZAS orthologues are widely conserved in land plants (Supplementary Data 2) and can be classified in at least 10 subgroups (Group1–Group10 in Supplementary Data 7), distributed in a taxon-dependent manner. Monocot ZASs build two different groups, Group 2 and Group 3, whereas ZASs of other species belong to only one subgroup (Supplementary Data 2). Since *O. sativa*, *Z. mays* and *S. bicolor* have both Group 2 and Group 3 orthologues in their genomes, it can be speculated that ZAS orthologues might have functionally differentiated in monocots. However, there are plant species, particularly *Brassicales* species, such as Arabidopsis, that do not contain ZAS orthologues (Supplementary Data 1 and Fig. 1b).

To confirm the ZAS activity in planta and explore the biological function of zaxinone, we characterized a retrotransposon *Tos17*-insertion mutant (NC0507)^30^ with an insertion in the tenth *ZAS* exon (Supplementary Fig. 6). First, we quantified zaxinone in roots and shoots of *zas* under normal conditions and upon phosphate (Pi)-deficiency, the latter causing a considerable increase in *ZAS* transcript levels in wild-type (Supplementary Fig. 7). Shoots of *zas* and wild-type seedlings showed similar contents of zaxinone and its precursor apo-10′-zeaxanthinal (Supplementary Fig. 3b), regardless of the Pi-supply. Under normal conditions, *zas* roots contained, compared to wild-type roots, significantly less zaxinone (12 versus 27 pg mg^−1^ dry weight; Fig. 1d) but more of other hydroxylated apocarotenoids, i.e. apo-10′-, apo-12′-, and apo-14′-zeaxanthinal (Supplementary Figure 3c). Exposure to Pi-starvation led to a general decrease of all measured zeaxanthin-derivatives, i.e. apo-10′-, apo-12′-, apo-14′-zeaxanthinal, and zaxinone, in both *zas* and wild-type roots (Fig. 1d; Supplementary Figure 3c). This decrease indicates that Pi-deficiency triggers the metabolization of these compounds. Here again, *zas* roots contained higher levels of apo-10′-, apo-12′-, and apo-14′-zeaxanthinal, but much less zaxinone (6 versus 16 pg mg^−1^ dry weight; Fig. 1d). In contrast to apo-10′-zeaxanthinal, amounts of the non-hydroxylated and non-substrate apocarotenoid β-apo-10′-carotenal were similar in *zas* and wild type seedlings and showed upon Pi starvation an increase rather than a decrease. These results are in agreement with in vitro data obtained for the ZAS substrate and products, are consistent with the expression pattern of this enzyme and demonstrate its role in zaxinone biosynthesis in planta. However, the remaining presence of zaxinone in *zas* plants suggests that ZAS activity is not the sole source of zaxinone. Indeed, rice genome encodes three ZAS orthologues, ZAS-L1-L3 (Fig. 1b), which could contribute to the zaxinone pool. Moreover, the presence of zaxinone in plants, such as Arabidopsis, which do not contain *ZAS* points to further, *ZAS*-independent biosynthesis route(s), which may involve different enzyme(s) or originate from attack of carotenoids by ROSs, as shown for cyclocitral^15^.

### Phenotypic characterization and rescue of *zas* mutant

**Fig. 2:**
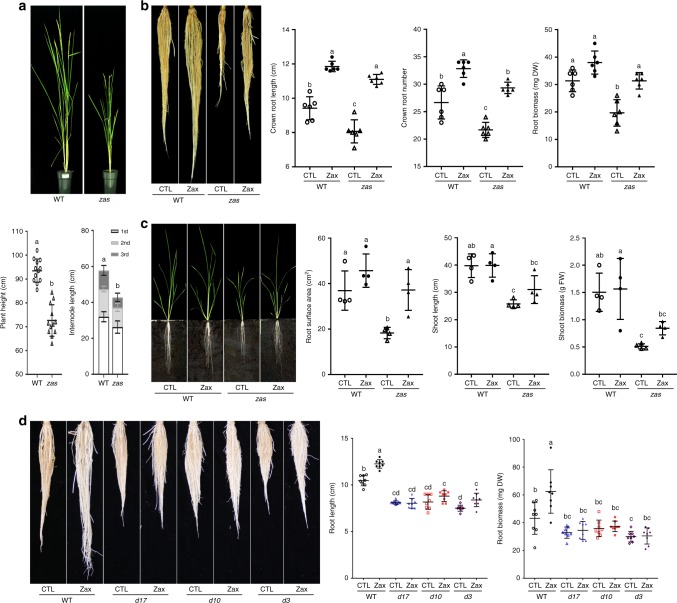
Phenotypic characterization and rescue of *zas* mutant. **a** Nipponbare wild-type and *zas* mutant plants at heading stage. **b** Roots of hydroponically grown wild-type and *zas* mutant seedlings in the absence (Control) and presence of zaxinone (2.5 µM). **c** Effect of zaxinone (10 µM) on soil-grown (rhizotron) wild-type and *zas* mutant plants. **d** Effect of zaxinone (2.5 µM) on root phenotype of Shiokari wild-type and *ccd* mutant seedlings grown hydroponically. Each data point represents one plant **(a** plant height *n* = 12, internode length *n* = 6; **b** *n* = 6; **c** *n* = 4; **d** *n* = 8). Data represent mean ± SD. Statistical analysis was performed using one-way analysis of variance (ANOVA) and Tukey’s post hoc test. Different letters denote significant differences (*P* < 0.05). CTL Control, Zax Zaxinone

Hydroponically grown *zas* seedlings showed reduction in main crown root length, number of crown roots, shoot length, and roots and shoots biomass (Fig. 2b, c), demonstrating a crucial role of ZAS in rice growth and development. Assuming that these phenotypes are caused by the observed lower roots zaxinone content, we supplied *zas* seedlings with exogenous zaxinone at a concentration of 2.5 μM for 3 weeks. This treatment rescued the crown root length, number, and roots biomass phenotypes (Fig. 2b). In soil, zaxinone application at 10 μM concentration increased the shoot length and biomass of *zas* mutant and rescued root phenotype (Fig. 2c). These data suggest that zaxinone is a positive regulator of growth in rice and is required at certain concentrations for normal development. Confirming this assumption, *zas* mutant showed generally reduced plant height in greenhouse (Fig. 2a). In field, we also observed a reduction in numbers of panicles, seeds, and tillers (Supplementary Fig. 8).

### Effect of zaxinone on SL biosynthesis

**Fig. 3:**
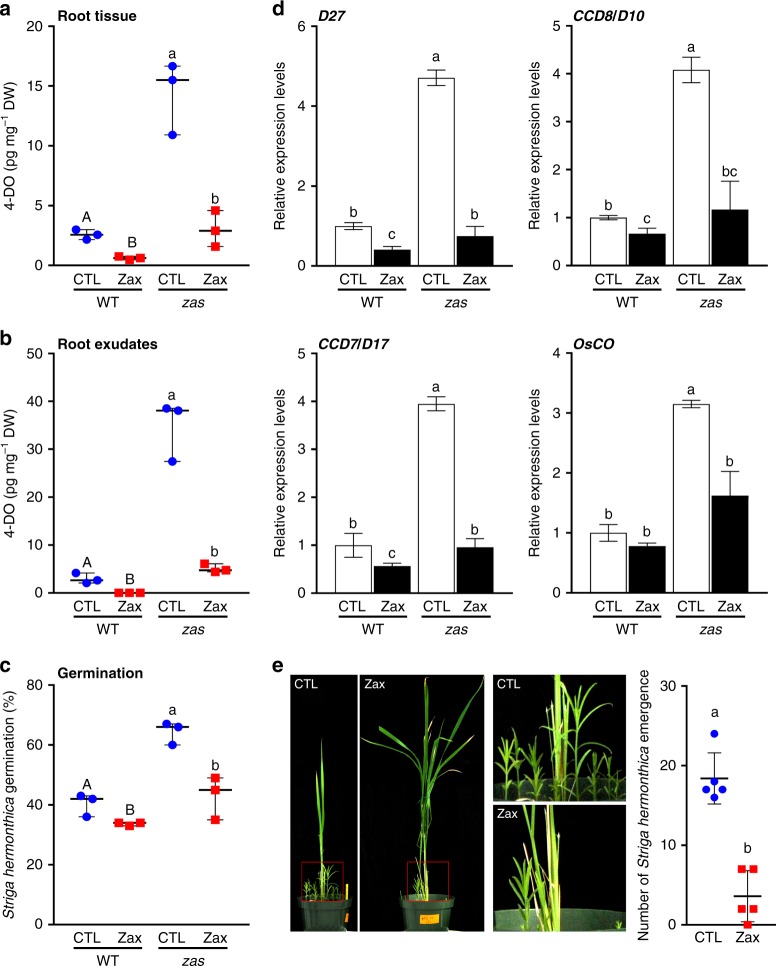
Effect of zaxinone on rice SL biosynthesis and release. **a, b** SL, 4-deoxyorobanchol (4-DO), quantification in wild-type and *zas* mutant roots (**a**) and root exudates (**b**) in response to zaxinone (5 µM) under Pi starvation. **c** *Striga* seeds germination rate upon treatment with exudates analyzed in **b**. **d** qRT-PCR analysis of transcript levels of SL biosynthesis genes (D27, CCD7, CCD8, and CO) transcript levels in root tissues analyzed in **a**. Transcript levels in wild-type control samples were normalized to 1. **e** Effect of zaxinone (10 µM) on *Striga* infestation of 6-weeks old rice cv. IAC-165 plants in soil. Effect on rice and *Striga* growth (Left, picture). Number of emerging *Striga* plants (Right). Bars represent mean ± SD; **a**–**d** *n* = 3 biological replicates; **e** *n* = 5 biological replicates; statistical analysis was performed using one-way analysis of variance (ANOVA) and Tukey’s post hoc test. Different letters denote significant differences (*P* < 0.05). CTL control, Zax zaxinone

Rice tillering is a complex developmental process governed by several plant hormones, including auxin, gibberellins, and SLs^31^. Taking into consideration that both zaxinone and SLs are derived from carotenoids, we hypothesized that the low tillering phenotype observed in the *zas* mutant (average 5 tillers in *zas* compared to 14 in wild-type; Supplementary Fig. 8c) may be related to a difference in SL production. SL levels has been associated with both high^5,6^ and low tillering phenotypes, as shown for the IAC-165 rice cultivar^32^. To test this hypothesis, we measured the SL (4-deoxyorobanchol) content in root tissues and exudates of hydroponically grown *zas* seedlings exposed to 1 week Pi-starvation, using LC-MS/MS. We also determined the *Striga* seed germinating activity of collected exudates, which usually correlates with the total amount of released SLs. Mutant roots and derived exudates thereof showed around 8–9 fold higher 4-deoxyorobanchol content than the wild-type samples (Fig. 3a, b). Consistent with this LC-MS- quantification, *zas* root exudates showed 20–30% higher *Striga* seed germination activity, compared to wild-type. Next, we exposed Pi-starved *zas* seedlings to zaxinone (5 μM) for 6 h and repeated SL measurements. This treatment reduced SL content in both roots (from 13 to 5 pg mg^−1^ dry-root-weight) and root exudates (from 35 to 5 pg mg^−1^ dry-root-weight) and lowered the germinating activity by 40% (Fig. 3c). Root exudates of *zas* seedlings under normal Pi supply also showed higher (increase by around 25%) germinating activity, compared to wild-type. Under these conditions, zaxinone application (5 μM) declined *zas* exudates germinating activity to a level below that of the wild-type sample (Supplementary Fig. 9a). Application of a higher concentration (10 μM) led to a more pronounced decrease in *Striga* seed germinating activity. Treatment of wild-type seedlings also led to a concentration-dependent reduction in *Striga* seed germinating activity of corresponding root exudates (Supplementary Fig. 9a). These data demonstrate that zaxinone is a negative regulator of rice SL biosynthesis and release. Next, we measured the transcript levels of the SL biosynthesis enzymes DWARF27 (D27), CCD7 (D17), CCD8 (D10), and the 4-deoxyorobanchol-forming carlactone oxidase (CO) in wild-type and *zas* seedlings exposed to Pi-starvation and upon treatment with zaxinone (5 μM). Under low Pi, the transcript levels of these genes were in *zas* mutant 2–3 fold higher than in wild-type (Fig. 3d). Zaxinone treatment decreased the transcript levels in *zas* mutant, but also in wild-type (Fig. 3d), which was already noticeable with a 2.5 μM concentration (Supplementary Fig. 10). These experiments demonstrate that zaxinone negatively regulates SL biosynthesis at transcript level under Pi-limiting conditions. Under normal conditions, we did not observe a reduction in transcript levels of SL biosynthesis enzymes upon zaxinone treatment (Supplementary Fig. 9b), which might be due to the very low level of the investigated transcripts. However, it is also possible that the down-regulation of SL biosynthesis by zaxinone under normal conditions, which is evidenced by the decrease in *Striga* seed germinating activity of corresponding root exudates (Supplementary Fig. 9a), is not mediated by decreasing transcript levels of the corresponding enzymes but by a different mechanism.

The effect of zaxinone on SL biosynthesis transcripts under Pi starvation might be a non-specific response. To exclude this possibility and to confirm the specificity of zaxinone’s effect, we measured the levels of several transcripts of ABA biosynthesis enzymes, and ABA and abiotic stress responsive genes in wild-type seedlings treated with zaxinone (5 µM) under phosphate starvation. As shown in (Supplementary Fig. 11a, b), application of zaxinone did not affect the level of these transcripts, indicating that this compound does not provoke a general stress response and suggests that its effect on SL transcript levels under Pi deficiency may be specific to SL biosynthesis.

The impact of zaxinone on SL biosynthesis and release, together with previously reported up-regulation of *ZAS* transcript in rice mycorrhizal roots^33^, suggests a possible role of this enzyme during mycorrhization. To test this hypothesis, we determined *ZAS* transcript levels in wild-type rice roots during different stages of colonization by the AM fungus *Rhizophagus irregularis. ZAS* transcript abundance in mycorrhizal roots increased at early and late stages of the colonization process: at 7 days, when the fungus is not yet colonizing the root, and at 35 days when the fungal arbuscules are abundant (Supplementary Fig. 12). This expression pattern partially mirrors root zaxinone content, which increases at 7 days post-inoculation (dpi) and decreases at 20 dpi, while at 35 dpi zaxinone content does not reflect ZAS up-regulation probably due to the occurrence of different factors, which may influence accumulation and stability of zaxinone in mycorrhizal roots. As observed in other mycorrhizal plants^34^, SL content shows highest values at early stage (7 dpi) and decreases to very low levels at later stages (20 and 35 dpi) of the colonization process (Supplementary Fig. 12b). The pattern of zaxinone and SLs on day 20 points out that SL levels during mycorrhization are not solely governed by zaxinone content, which is expected because SL levels are likely also controlled by further factors, such as other plant hormones. Overall these data indicate a complex interplay between zaxinone and SLs during mycorrhization.

Next, we investigated the mycorrhization susceptibility of the *zas* mutant, using two phylogenetically diverse AM fungi (*R. irregularis* and *Funneliformis mosseae)*. Compared to wild-type, *zas* plants showed much lower colonization with both fungi. Accordingly, transcripts of the AM inducible Pi-transporter, used as symbiosis marker, were barely detectable in *zas* roots (Supplementary Fig. 13). However, the lack of *ZAS* did not impact the phenotype of the arbuscules that appeared well developed and regularly branched, as observed in confocal microscope images of WGA-stained wild-type and *zas* mycorrhizal roots (Supplementary Fig. 13e). These results demonstrate that *ZAS* does not affect the fungal morphology, is not essential for establishing AM symbiosis, but is required for optimal colonization levels.

Phylogenetic analysis revealed that ZAS orthologues are conserved in land plant species from mosses to higher plants. Remarkably, ZAS genes have been lost during evolution in non-AM plants, such as *Brassicaceae* species, including *A. thaliana* characterized by the loss of many symbiotic genes^35^. Based on the lower colonization level of the *zas* mutant, it is tempting to assume a correlation between loss events of ZAS gene(s) and AM symbiosis. To test this hypothesis, we conducted CCD gene survey in 16 genomes of non-AM host plants including moss, gymnosperm, and angiosperm species (Supplementary Data 3; Supplementary Data 8). The results obtained demonstrate that all the non-AM host plants considered have lost ZAS orthologues, while orthologous of other CCD types have remained well conserved. Our results suggest that the loss of ZAS orthologues was a common evolutionary event in all investigated non-AM host plants. However, we also identified seven AM host plant species that have lost ZAS orthologues. It can be hypothesized that these species may have developed alternative mechanisms to compensate the lack of ZAS to sustain successful AM symbiosis.

Finally, to test whether the zaxinone effect requires the F-box protein D3 involved in SL-dependent negative feedback regulation of SL biosynthesis, we treated Pi-starved *d3* mutant and corresponding wild-type seedlings with zaxinone and the SL analog GR24, used as a control, for 6 h. Zaxinone application lowered the SL content in *d3* roots and root exudates, and decreased SL transcript levels in roots of both wild-type and *d3* seedlings (Supplementary Fig. 14a, b). In contrast, *rac*-GR24 affected the transcript levels only in wild-type seedlings (Supplementary Fig. 14c). These results show that D3 is not required for zaxinone signal transduction. To test whether the growth promoting activity of zaxinone is independent of SLs, we applied the compound to SL biosynthesis and perception mutants. Interestingly, zaxinone application did not promote the growth of roots in SL deficient mutants (Fig. 2d), which indicates that the growth promoting effect of zaxinone likely requires functional SL biosynthesis. Apart from the potential application of zaxinone to increase crop growth, this compound might be also utilized to combat root parasitic weeds. To test this possibility, we applied zaxinone (10 μM) to *Striga* susceptible rice cv. IAC-165 plants grown in pots with *Striga* infested soil. This treatment clearly reduced (by around 80%) the number of emerging *Striga* plants, compared to the untreated control (Fig. 3e).

## Discussion

In summary, we identified zaxinone as a member of the ubiquitous family of carotenoid-derived signaling molecules and hormones. Zaxinone regulates the growth and development of rice and controls the level of SLs, which are key regulators of rice architecture, biotic and abiotic stress responses, and major components of the communication process with beneficial symbiotic partners and harmful parasitic invaders. Besides the potential applications for increasing rice growth and combating *Striga*, increased knowledge of zaxinone may help us to better understand rice development, land plant evolution and the plant-AM fungi interplay underlying a symbiosis taking place in the majority of land plants.

## Methods

### Plant material and growth conditions

Rice plants were grown under controlled conditions (day/night temperature of 28/22 ^o^C and a 12 h photoperiod, 200 µmol photons m^−2^ s^−1^). Rice seeds were surface-sterilized in 50% sodium hypochlorite solution with 0.01% Tween-20 for 20 min. The seeds were rinsed with sterile water and germinated in the dark. The pre-germinated seeds were transferred to petri dishes containing half-strength liquid Murashige and Skoog (MS) medium and incubated in a percival for 7 days.

For phenotyping of *zas* mutant, the seedlings were transferred into pots filled with soil containing half-strength modified Hoagland nutrient solution. The nutrient solution consisted of 5.6 mM NH_4_NO_3_, 0.8 mM MgSO_4_^.^7H_2_O, 0.8 mM K_2_SO_4_, 0.18 mM FeSO_4_^.^7H_2_O, 0.18 mM Na_2_EDTA^.^2H_2_O, 1.6 mM CaCl_2_^.^2H_2_O, 0.8 mM KNO_3_, 0.023 mM H_3_BO_3_, 0.0045 mM MnCl_2_^.^4H_2_O, 0.0003 mM CuSO_4_^.^5H_2_O, 0.0015 mM ZnCl_2_, 0.0001 mM Na_2_MoO_4_^.^2H_2_O, and with or without 0.4 mM K_2_HPO_4_^.^2H_2_O, resulting in the +Pi and -Pi conditions, respectively. The pH of the solution was adjusted to 5.8, and the solution was applied every third day. On day 56, phenotypic data were recorded.

For rescuing *zas* mutant phenotypes and investigating the effect of zaxinone on different genotypes, 1 week-old seedlings were grown hydroponically in Hoagland nutrient solution containing K_2_HPO_4_^.^2H_2_O (+Pi), 2.5 µM zaxinone (solved in 0.1% acetone) or the corresponding volume of the solvent (control) for 3 weeks. The solution was changed every other day, adding the chemical at each renewal.

For rescuing *zas* mutant phenotypes in the rhizotron system (48 cm × 24 cm × 5 cm), 3 days-old seedlings were grown in soil with Hoagland nutrient solution containing 0.4 mM K_2_HPO_4_^.^2H_2_O (+Pi) and10 µM zaxinone for 2 weeks. The solution was changed every other day, adding the chemical at each renewal. To increase zaxinone stability, 1 µl/ml emulsifier (cyclohexanone + Atlas G1086 kindly provided by Mr. Han Rieffe of CRODA, Gouda; The Netherlands) was added into Hoagland nutrient solution. Root surface area was analyzed with the ImageJ software.

For transcript analysis, 1 week-old seedlings were grown hydroponically in half-strength modified Hoagland nutrient solution with K_2_HPO_4_^.^2H_2_O ( + Pi) and without K_2_HPO_4_^.^2H_2_O (-Pi) for 7 days. Seedlings were further treated with zaxinone for 6 h, and tissues were collected.

For SL analysis, 1 week-old seedlings were transferred into 50 ml falcon tubes (three seedlings per tube), containing Hoagland nutrient solution with K_2_HPO_4_^.^2H_2_O (+Pi), and grown in the growth cabinet for 1 week. Rice seedlings were then subjected to phosphate deficiency (-Pi) for another 1 week. On the day of root exudates collection, rice seedlings were first treated with 5 µM zaxinone for 6 h, and then root exudates and root tissues were collected from each tube for LC-MS/MS analysis and *Striga* bioassays.

Synthetic zaxinone was purchased (customized synthesis) from Buchem B.V. (Apeldoorn, The Netherlands).

### Qualitative and quantitative analysis of zaxinone

For the identification and quantification of zaxinone *in planta*, plant material was lyophilized and grinded. Twenty five milligrams of tissue powder, spiked with 1.65 ng of D_3_-zaxinone (customized synthesis; Buchem B.V., Apeldoorn, The Netherlands), was extracted with 2 ml of ethyl acetate twice, by sonication for 15 min in an ultrasonic bath followed by centrifugation for 8 min at 1356 × *g* at 4 °C. The two supernatants were combined and dried under vacuum. The dried extract was dissolved in 100 µl of ethyl acetate and 2 ml of hexane prior the following purification. The sample was run through a Silica gel SPE column (500 mg/3 ml) preconditioned with 3 ml of ethyl acetate and 3 ml of hexane. After washing with 3 ml hexane, zaxinone were eluted in 3 ml ethyl acetate and evaporated to dryness under vacuum. The residue was re-dissolved in 200 μl of acetonitrile:water (25:75, v:v) for zaxinone, and filtered through a 0.22 μm filter for LC-MS/MS analysis. Qualitative analysis of zaxinone extracted from plant material was performed on a Dionex Ultimate 3000 UHPLC system coupled with a Q-Exactive plus MS (Thermo Scientific) with a heated-electrospray ionization source. Chromatographic separation was carried out on a Phenomenex Gemini C_18_ column (150 × 2.0 mm, 5 μm) with the mobile phase of water: acetonitrile: formic acid (95:5:0.1, v:v:v, A) and acetonitrile: formic acid (100:0.1, v:v, B) in a gradient program (0–20 min, 25–100 % B, followed by washing with 100 % B and equilibration with 25 % B). The flow rate was 0.2 ml/min. The injection volume was 10 μl, and the column temperature was maintained at 35 °C for each run. The MS conditions were as follows: positive mode, spray voltage of 4.0 kV, auxiliary gas heater temperature of 310 ^o^C, sheath gas flow rate of 30 arbitrary units, auxiliary gas flow rate of 10 arbitrary units, capillary temperature of 320 ^o^C, S-lens RF level of 55, resolution of 70,000, and NCE of 15 eV for MS/MS. The quantification of zaxinone in rice tissues was performed by using HPLC-Q-Trap-MS/MS with MRM mode. Chromatographic separation was achieved on an Acquity UPLC BEH C_18_ column (50 × 2.1 mm; 1.7 μm; Waters) with mobile phases consisting of water: acetonitrile (95:5, v:v, A) and acetonitrile (B), both containing 0.1% formic acid, and the following linear gradient (flow rate, 0.2 ml/min): 0–20 min, 25–100 %, followed by washing with 100 % B and equilibration with 25 % B. The injection volume was 5 μl, and the column temperature was maintained at 30 °C for each run. The MS parameters were as follows: positive ion mode, ion source of turbo spray, ion spray voltage of 5500 V, curtain gas of 40 psi, collision gas of medium, gas 1 of 60 psi, gas 2 of 50 psi, turbo gas temperature of 400 °C, declustering potential of 60 V, entrance potential of 10 V, collision energy of 20 eV, collision cell exit potential of 10 V. The characteristic MRM transitions (precursor ion → product ion) were 275 → 257, 275 → 239, 275 → 173 for zaxinone; 278 → 260, 278 → 242, 278 → 173 for D_3_-zaxinone.

### Quantitative analysis of 4-DO in root exudates and tissues

For the quantification of 4-deoxyorobanchol in rice root exudates, 50 ml of root exudates spiked with 0.672 ng of D_6_–5-deoxystrigol, was brought on a 500 mg/3 ml fast SPE C_18_ column preconditioned with 6 ml of methanol and 6 ml of water. After washing with 6 ml of water, SLs were eluted with 5 ml of acetone. The 4-deoxyorobanchol fraction (acetone-water solution) was concentrated to SL aqueous solution (∼1 ml), followed by the extraction with 1 ml of ethyl acetate. 750 μl of 4-deoxyorobanchol enriched organic phase was then transferred to 1.5 ml tube and evaporated to dryness under vacuum. The dried extract was dissolved in 100 μl of acetonitrile: water (25:75, v:v) and filtered through a 0.22 μm filter for LC-MS/MS analysis.

For the quantification of 4-deoxyorobanchol in rice root, plant tissue material was lyophilized and grinded. 30 mg root tissue spiked with 0.672 ng of D_6_–5-deoxystrigol was extracted with 2 ml of ethyl acetate in an ultrasound bath (Branson 3510 ultrasonic bath) for 15 min, followed by centrifugation for 8 min at 1356 × *g* at 4 ^o^C. The supernatant was collected and the pellet was re-extracted with 2 ml of ethyl acetate. Then the two supernatants were combined and dried under vacuum. The residue was dissolved in 100 µl of ethyl acetate and 2 ml of hexane. The resulting extract solution was loaded on a Silica gel SPE column (500 mg/3 ml) preconditioned with 3 ml of ethyl acetate and 3 ml of hexane. After washing with 3 ml of hexane, 4-deoxyorobanchol was eluted in 3 ml of ethyl acetate and evaporated to dryness under vacuum. The residue was re-dissolved in 200 μl of acetonitrile: water (25:75, v:v) and filtered through a 0.22 μm filter for LC-MS/MS analysis. 4-deoxyorobanchol was analyzed by using HPLC-Q-Trap-MS/MS with MRM mode. Chromatographic separation was achieved on an Agilent 1200 HPLC system with an Acquity UPLC BEH C_18_ column (50 × 2.1 mm; 1.7 μm; Waters). Mobile phases consisted of water: acetonitrile (95:5, v:v, A) and acetonitrile (B), both containing 0.1 % formic acid. A linear gradient was optimized as follows (flow rate, 0.2 ml/min): 0–10 min, 25 to 100 % B, followed by washing with 100% B and equilibration with 25% B. The injection volume was 5 μl and the column temperature was maintained at 30 °C for each run. The MS parameters were listed as follows: positive ion mode, ion source of turbo spray, ion spray voltage of 5500 V, curtain gas of 20 psi, collision gas of medium, gas 1 of 80 psi, gas 2 of 70 psi, turbo gas temperature of 400 °C, declustering potential of 60 V, entrance potential of 10 V, collision energy of 20 eV, collision cell exit potential of 15 V. The characteristic MRM transitions (precursor ion → product ion) were 331 → 216, 331 → 97 for 4-deoxyorobanchol; 337 → 222, 337 → 97 for D_6_–5-deoxystrigol.

### Quantitative analysis of rice apocarotenoids

Quantitative analysis of apocarotenoids was carried out as the method described^36^. Approximate 20 mg tissue powder spiked with internal standard (IS) mixture (1 ng each standard) was extracted with 2 ml of MeOH containing 0.1% BHT by sonication for 15 min in an ultrasound bath (Branson 3510 ultrasonic bath), followed by centrifugation for 8 min at 1356 × *g* at 4 °C. The supernatant was collected and the pellet was re-extracted with 1 ml of the same solvent. Then, the two supernatants were combined and dried under vacuum. The residue was re-dissolved in 150 μl of acetonitrile and filtered through a 0.22 μm filter for LC-MS analysis. APOs analysis was performed on a Dionex Ultimate 3000 UHPLC system coupled with a Q-Orbitrap- MS (Q-Exactive plus MS, Thermo Scientific) with a heated-electrospray ionization source. Chromatographic separation was carried out on an ACQUITY UPLC BEH C_18_ column (100 × 2.1 mm, 1.7 μm) with an UPLC BEH C_18_ guard column (5 × 2.1 mm, 1.7 μm) maintained at 35 ^o^C. The mobile phases A (H_2_O/ACN/FA, 80/20/0.1, v/v/v) and B (ACN/IPA/FA, 60/40/0.1, v/v/v) were used following the gradient program: 0–1 min, 0% B; 1–3 min, 0% B to 40% B; 3–8 min, 40% B to 80% B; 8–14 min, 80% B to 90% B; 14–15 min, 90% B to 100% B; followed by washing with 100% B and equilibration with 100% A. The flow rate was 0.2 ml/min, and the injection volume was 5 µl. The MS parameters were as follows: sheath gas flow rate of 40 arbitrary units, auxiliary gas flow rate of 20 arbitrary units, spray voltage of 4.0 kV, capillary temperature of 350 ^o^C, auxiliary gas heater temperature of 250 ^o^C, S-lens RF level of 50, and resolution of 280,000.

### In vitro assays

Full-length *ZAS* cDNA was produced from total rice RNA by RT-PCR. The cDNA was then amplified, lacking the stop codon, and ligated into pBAD/Thio-TOPO (Invitrogen), enabling the expression of the enzyme fused with thioredoxin at the N-terminus and equipped with a C-terminal 6-His-tag. Integrity of the obtained plasmid pThio-ZAShis was confirmed by sequencing. The fusion protein was expressed in *E. coli* BL21 cells transformed with the plasmid pGro7 (Takara Bio Inc.; Mobitec, Göttingen, Germany) that enables co-expression of the groES-groEL-chaperone system. Bacterial growth, induction, and preparation of soluble supernatants were carried out as method described^16^. To study the enzymatic activity of ZAS, we incubated the soluble supernatants of overexpressing *E. coli* cells with the following carotenoids: β-carotene, 9-*cis*-β-carotene, zeaxanthin and lycopene. We also tested 3-OH-β-apo-10′-carotenal, 3-OH-β-apo-8′-carotenal, 3-OH-β-apo-12′-carotenal, β-apo-10′-carotenal, β-apo-8′-carotenal, 9-*cis*-α-apo-10′-carotenal, 3-OH-9-*cis*-α-apo-10′-carotenal, 9-*cis*-β-apo-10′-carotenal, 3-OH-9-*cis*-β-apo-10′-carotenal, apo-10′-lycopenal, and apo-8′-lycopenal (Supplementary Fig. 1). Assays were prepared, performed, and extracted according to the protocol^16^. β-carotene, 9-*cis*-β-carotene, zeaxanthin, and lycopene were purchased from Sigma-Aldrich. Synthetic apocarotenoids were purchased from Buchem B. V. (customized synthesis, Apeldoorn; The Netherlands). For conversion rate measurements, in vitro assays were stopped by freezing in liquid nitrogen at nine different time points during 2 h of incubation and extracted as method described^16^. In vitro assays sample spiked with 60 µM retinal (internal standard, purchased from Buchem B. V., Apeldoorn; The Netherlands), was extracted as described^16^ for UHPLC-DAD analysis. The quantification of the product, zaxinone, was performed by calculating peak areas at maximum absorption wavelength, using a standard curve. Analysis of ZAS in vitro assays sample was performed on Ultimate 3000 UHPLC system with a YMC Carotenoid C_30_ column (250 × 4.0 mm, 5 µm) at a flow rate of 0.6 ml/min and a column temperature of 30 ^o^C. Mobile phases included methanol: tert-butylmethyl ether (1:1, v:v, A) and methanol: water:tert-butylmethyl ether (30:10:1, v:v:v, B). The gradient started from 100% B to 45% B in 15 min followed by isocratic elution with 45% B for 9 min.

To detect the expected C_9_-dialdehyde product, C_9_-dialdehyde was identified by using LC-MS after derivatization with *O*-(2,3,4,5,6-pentafluorobenzyl)hydroxylamine hydrochloride. Briefly, C_9_-dialdehyde was extracted with 200 μl of chloroform: methanol (2:1, v:v). The extract was dried under vacuum and re-dissolved in 100 μL of methanol, followed by the derivatization with 50 μl of *O*-(2,3,4,5,6-pentafluorobenzyl)hydroxylamine hydrochloride (25 mg ml^−1^ in MeOH) at 35 °C for 1 h. The C_9_-dialdehyde derivative was dried and re-dissolved in 50 μl of DMSO for LC-MS analysis. Analysis was performed on a Dionex Ultimate UHPLC system coupled with a Q-Exactive plus MS. Chromatographic separation was carried out on a Phenomenex Gemini C_18_ column (150 × 2.0 mm, 5 μm) with the mobile phase of acetonitrile: water: formic acid (95:5:0.1, v:v:v, A) and acetonitrile: water: formic acid (5:95:0.1, v:v:v, B) at the flow rate of 0.2 ml/min and the column temperature of 35 ^o^C. The gradient was as follows: 0–20 min, 50–100% B, followed by washing with 100% B and equilibration with 50% B. The MS parameters were as follows: positive mode, spray voltage of 4.0 kV, auxiliary gas temperature of 310 ^o^C, sheath gas flow rate of 30 arbitrary units, auxiliary gas flow rate of 10 arbitrary units, capillary temperature of 320 ^o^C, S-lens RF level of 55, resolution of 70,000, and NCE of 15 eV.

### Recombinant protein expression and purification

For purified maltose-binding protein (MBP)-fusion protein, full-length *ZAS* cDNA was amplified with PCR using primers (Supplementary Data 4) and cloned into the pET-His6 MBP N10 TEV LIC cloning vector (2C-T vector; http://www.addgene.org/29706/) with 6xHis and MBP tags at the N-terminus^37^. After sequence confirmation (KAUST, Bioscience Core Lab), plasmid was then transformed into *E. coli* BL21 (DE3) cells. The cells were grown in LB broth containing ampicillin (100 mg/ml) at 37 °C until an OD600 of 0.5 and expression was induced with 0.1 mM isopropyl-β-D-thiogalactopyranoside (IPTG) at 16 °C for 16 h under shaking. Harvested cells were resuspended in lysis buffer: 50 mM Tris–HCl (pH 8.0), 200 mM NaCl, detergent (0.5% Triton X-100), and 2 mM DTT. After sonication (40% amplitude (10 min), 2 s ON, 1 s OFF) on ice for 10 mins, the lysate were centrifuged at 74,766 × *g* for 30 min at 4 °C. Further, the supernatant was allowed to bind to MBP-beads-Amylose Resin (New England BioLabs) for 2 h at 4 °C. Then, washed three times with buffer (50 mM Tris–HCl (pH 8.0), 200 mM NaCl, 2 mM DTT) for 15 min each at 4 °C and eluted with 50 mM maltose monohydrate after shaking for 30 min at 4 °C. The eluted protein was further used for in vitro assays described previously^16^.

### *Striga hermonthica* seed germination bioassays

*Striga* seed germination bioassay was carried out according to the protocol^38^. After 10 days of pre-conditioning (30 ^o^C under moist for 10 days), *Striga* seeds were supplied with 50 μl of root exudates of zaxinone treated and non-treated wild-type and *zas* mutant. After application, *Striga* seeds were incubated at 30 ^o^C in dark for 2 days. Germinated (seeds with radicle) and non-germinated seeds were counted under a binocular microscope to calculate germination rate (%).

### *Striga hermonthica* infection in rice

About 10 mg (~4000) *Striga* seeds were thoroughly mixed in 2 L  of soil and sand mixture (1:1), added in a 3 L  plastic pot and kept under moisture conditions at 35 ^o^C for 10 days. Then three 5 days-old rice seedlings (*Oryza sativa* L. cv. IAC-165) were planted in the middle of each *Striga* pot. A stock of 10 mM of zaxinone was prepared by mixing with an emulsifier (cyclohexanone + Atlas G1086) kindly provided by Mr. Han Rieffe of CRODA, Gouda; The Netherlands). After 3 days of rice planting, zaxinone was applied at 10 μM concentration in 20 ml water in each pot. Zaxinone was applied twice a week for 5 weeks. *Striga* emergence were recorded after 6 weeks of rice planting.

### Gene expression analysis

Total RNA was extracted from roots and shoots using a Qiagen Plant RNeasy Kit according to the manufacturer’s instruction (Qiagen, Hilden; Germany). cDNA was synthesized from 1 µg of total RNA using iScript cDNA Synthesis Kit (BIO-RAD Laboratories, Inc, 2000 Alfred Nobel Drive, Hercules, CA; USA) according to the instructions in the user manual. For mycorrhizal samples single-strand cDNA was synthesized from 1 µg of total RNA using Super-Script II (Invitrogen) according to the instructions in the user manual. Primers used for real-time quantitative RT-PCR (qRT-PCR) analysis are listed in (Supplementary Data 4). qRT-PCR was performed using SYBR Green Master Mix (Applied Biosystems; www.lifetechnologies.com) in a StepOnePlus (Life Technologies, Carlsbad, CA, USA). For mycorrhizal samples qRT-PCR was performed using a Rotor-Gene Q 5plex HRM Platform (Qiagen, Hilden; Germany). The 2-^ΔΔ^*C*_T_ method was used to calculate the relative gene expression levels^39^ and rice Ubiquitin (*OsUBQ* or *OsRubQ1*) gene (Supplementary Data 4) was used as the internal control to normalize target gene expression.

### Statistical analysis

All of the experiments were performed with at least three biological replicates each. Statistical tests were carried out through one-way analysis of variance (one-way ANOVA) and Tukey’s post hoc test or via two-tailed Student’s *t*-tests, using a probability level of *P* < 0.05. All statistical elaborations were performed using R program or PAST statistical package version 2.16^40^.

### Survey of CCD orthologues in plants

We obtained 68 land plant genomes (protein fasta files) from in NCBI RefSeq assembly and one genome from NCBI Genbank assembly. Information regarding genome assemblies used in this study is described in Supplementary Data 1. Obtained protein sequences were annotated were by Pfam-A data base (1) using Hmmscan program included in HMMER v3.1b1 package (http://www.hmmer.org/). We then extracted protein sequences annotated as PF03055 (Retinal pigment epithelial membrane protein). The obtaining sequences were filtered with respect to their length of PF03055 regions (≥ 400 aa). We then checked position of the sequences on their species genomes and removed transcript variants (the longest one was used for the analysis). We obtained 783 sequences after the filtration and used them as CCD orthologues for the phylogenetic analysis.

### Phylogenetic analysis

We obtained the sequence alignment of CCD orthologues using MAFFT ver. 3 (2) with default settings. Neighbour-joining trees were constructed from the aligned sequences with MEGA7 (3). Bootstrap values were generated from 100 pseudoreplicates using MEGA7. We extracted 106 sequences belonging to *ZAS* subcluster in the resultant tree. We re-constructed the Neighbour-joining tree of *ZAS* orthologues using the same method as described above. We also constructed Neighbour-joining tree using *O. sativa* and *A. thaliana* sequences only. Tree construction was performed with the same methods described above.

To investigate the presence of *ZAS* orthologues in non-host plants of arbuscular mycorrhizal fungi (non-host AM plants), we conducted CCD gene survey using genomes of non-AM plants analyzed, as described previously^41^ (Supplementary Data 3). Genomes (protein fasta files) with accession numbers including “GCF_” prefix were obtained from RefSeq genome assembly database. Protein fasta files of *Picea abies* were obtained from ConGenIE (Conifer Genome Integrative Explorer) web resource (http://congenie.org). Protein fasta files of *Dianthus caryophyllus* was retrieved from canation.kazusa.or.jp. Genome fasta files (Scaffold or contig levels) of *Utricularia gibba* (GCA_002189035.1), *Spirodela polyrhiza* (GCA_001981405.1) were retrieved from GenBank database. As protein fasta files were not prepared for these species, we conducted gene prediction from these genome assemblies with AUGUSTUS 3.3.1^42^. RNAseq sequences (SRR3090696 and SRR3090697 of *S. polyrhiza* and SRR5046448 of *U. gibba*) were used to generate hint files for the prediction with AUGUSTUS, following the developer’s instructions. CCD sequences of *Spirodela polyrhiza* and *Utricularia gibba* predicted in this study were shown in (Supplementary Data 5). We also evaluated the qualities of genome sequence data used in this study with BUSCO software using protein fasta files^43^.

### Field trial

Field experiment was carried out at the experimental farm of CREA-RIS (Consiglio per la ricerca in agricoltura e l’analisi dell’economia agraria, Unità di ricerca per la risicoltura) located in Northern Italy (45°19′17″ N 8°25′11″E). Seeds of wild-type (cv. Nipponbare) and *zas* mutant were germinated in soil. During the cropping season (May–October), irrigation treatments by flushing was provided from July (panicle initiation) to September (late flowering), taking into consideration climatic conditions and rainfalls. In detail, the irrigation treatment consisted of an input of water to reach the complete submersion of the field; after the complete flooding, the flux of water was interrupted. Plot size was 2 m × 1 m. Three treatments of organic N (12.5%) were applied before sowing (May), during tillering (June), and one in July (panicle initiation). Phenotyping of wild-type and *zas* shoot, including node and spike length, number of tillers, panicle, and seed number, was performed in October, immediately before harvest. For each genotype 12 plants were considered.

### Plant and fungal materials for mycorrhizal experiment

Seeds of wild-type (cv. Nipponbare) and *zas* mutant were germinated in pots containing sand and incubated for 10 days in a growth chamber under a 14 h light (23 °C)/10 h dark (21 °C). Plants used for mycorrhization were inoculated with *Funelliformis mosseae* (BEG 12, MycAgroLab, France) or *Rhizophagus irregularis* (INOQ GmbH, Germany). Both fungi inoculum (25 %) were mixed with sterile quartz sand and used for colonization. Plants were watered with a modified Long-Ashton (LA) solution containing 3.2 μM Na_2_HPO_4_·12H_2_O^44^ and were grown in a growth chamber under 14 h light (24 °C)/10 h dark (20 °C) regime. In the time course experiment wild-type and *zas* plants inoculated with *R. irregularis* were sampled at 7, 20, 29, and 35 dpi.  Wild-type and *zas* mycorrhizal roots were stained with 0.1% cotton blue in lactic acid and the estimation of mycorrhizal parameters was performed as the method^45^ using MYCOCALC (http://www2.dijon.inra.fr/mychintec/Mycocalc-prg/download.html). For the molecular analyses, roots were immediately frozen in liquid nitrogen and stored at −80 °C.

### Assessment of the arbuscule phenotype

Roots of wild-type and *zas* plants were embedded in agarose (8%). Agarose blocks were cut into 180 μm vibratome slices, which were put on slides. Slices were treated for 5 min in 0.5% commercial bleach diluted in Pi buffer (pH 7), washed again, and then inocubated for 2 h with wheat germ agglutinin-fluorescein isothiocyanate (WGA-FITC) (Sigma-Aldrich) at final concentration of 10 μg ml^−1^, to detect the chitin of fungal cell walls. Working conditions for the Leica TCS SP2 confocal microscope (Leica Microsystem GmbH, Wetzlar, Germany) for observation and image acquisition were performed according to the protocol^46^.

### Reporting Summary

Further information on experimental design is available in the Nature Research Reporting Summary linked to this article.

## Supplementary information

Supplementary Information

Description of Additional Supplementary Files

Supplementary Data 1

Supplementary Data 2

Supplementary Data 3

Supplementary Data 4

Supplementary Data 5

Supplementary Data 6

Supplementary Data 7

Supplementary Data 8

Source Data

Reporting Summary

## Data Availability

Materials are available under an MTA agreement with the King Abdullah University of Science and Technology (KAUST). All data generated or analyzed during this study are included in this published article and its supplementary information files. The Source Data underlying Figs. 1d, 2a–d, and 3a–e, and Supplementary Figures 2a, 3, 6c, 7, 8, 9ab, 10, 11, 12a–d, 13a–d and 14a–c are provided as a Source Data file.
