# Supplementary material for: The apocarotenoid metabolite zaxinone regulates growth and strigolactone biosynthesis in rice
Source: Nat Commun. 2019 Feb 18;10:810. doi: 10.1038/s41467-019-08461-1 (PMC6379432; doi:10.1038/s41467-019-08461-1)
Supplement: Supplementary file 1 — Supplementary Information [file 41467_2019_8461_MOESM1_ESM.pdf]

SUPPLEMENTARY INFORMATION

The Apocarotenoid Metabolite Zaxinone Regulates Growth and Strigolactone Biosynthesis in Rice

Jian You Wang, Imran Haider, Muhammad Jamil, Valentina Fiorilli, Yoshimoto Saito, Jianing Mi, Lina Baz, Boubacar A. Kountche, Kun-Peng Jia, Xiujie Guo, Aparna Balakrishna, Valentine Otang Ntui, Beate Reinke, Veronica Volpe, Takashi Gojobori, Ikram Blilou, Luisa Lanfranco, Paola Bonfante, Salim Al-Babili[1]*

[1]King Abdullah University of Science and Technology, Division of Biological and Environmental Science and Engineering, the BioActives Lab, Thuwal, 23955-6900, Saudi Arabia.

*email: salim.babili@kaust.edu.sa

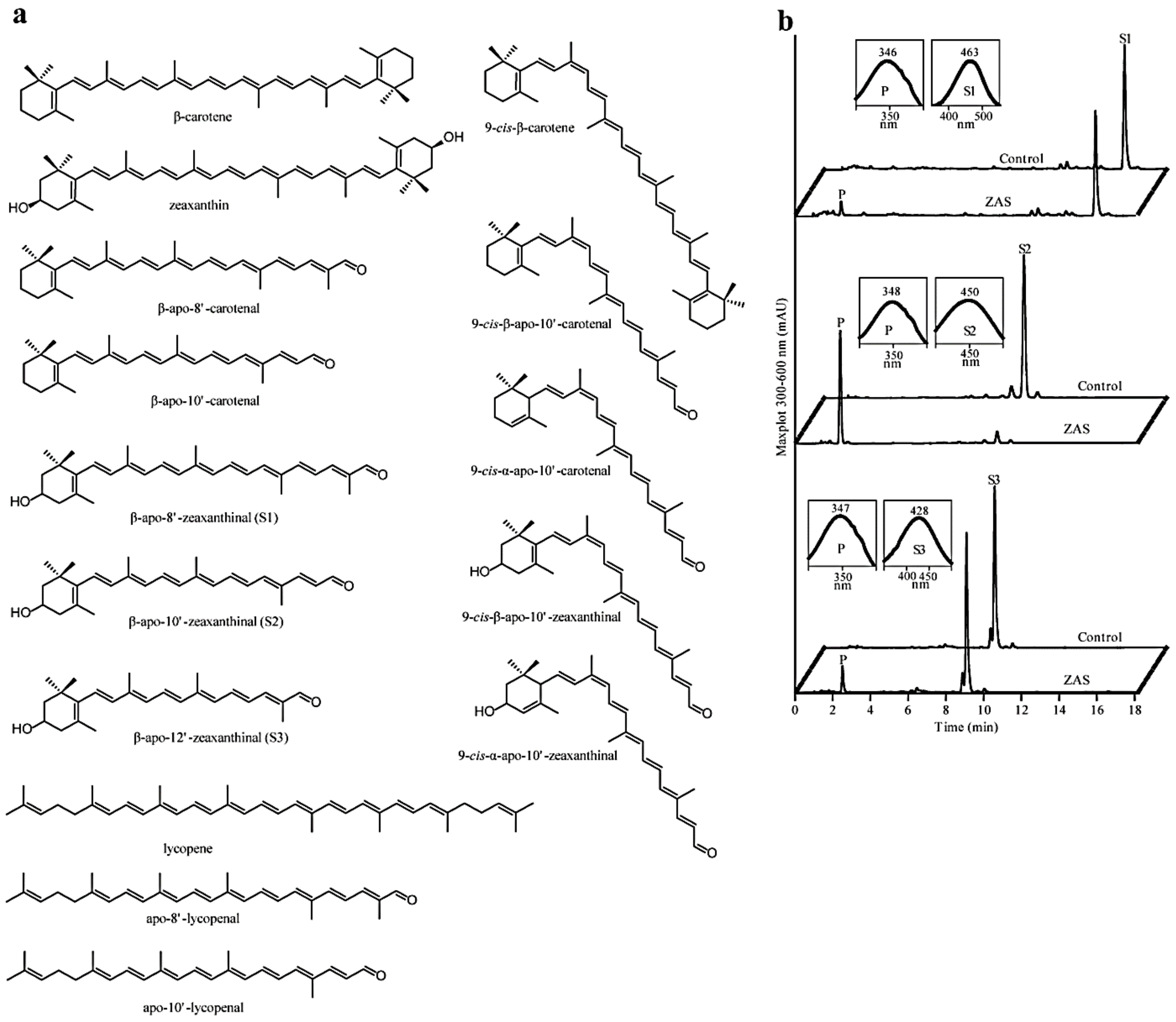

Supplementary Figure 1 | UHPLC analysis of ZAS incubation with carotenoids and apocarotenoids. (a) Structures of carotenoids and apocarotenoids used as substrates in ZAS *in vitro* assays. **(b)** The enzyme converted only three of the tested substrates, i.e. apo-8'-zeaxanthinal (S1), apo-10'-zeaxanthinal (S2), and apo-12'-zeaxanthinal (S3), into zaxinone (P). The UV-visible spectra of the substrates (S1, S2, and S3) and product (P) are shown in the insets, mAU, arbitrary units.

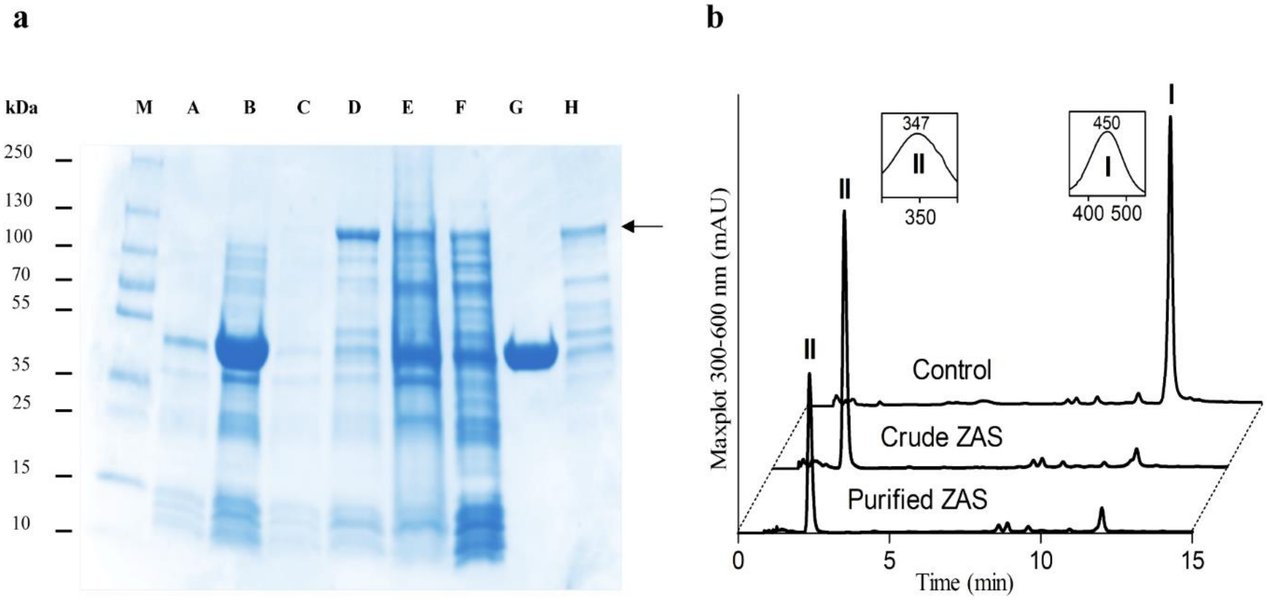

Supplementary Figure 2 | UHPLC analysis of purified ZAS incubation with apo-10'-zeaxanthinal. (a) Coomassie Blue-stained SDS/PAGE gel analysis of maltose binding protein (MBP)-ZAS purification fractions. Lanes represent: **M**, prestained protein molecular weight marker (size in kDa); total protein extract of control cells, an aliquot corresponding to 100 µl culture, before **A** and after induction with 0.1 mM IPTG **B**, total protein extract of cells, an aliquot corresponding to 100 µl culture, producing MBP-ZAS before **C** and after induction **D**; **E**, fraction of total soluble protein of MBP-ZAS producing cells, an aliquot corresponding to 150 µl culture; **F**, flow through fraction of **E** after binding to amylose, an aliquot corresponding to 150 µl culture; **G**, eluate from amylose resin load with control cell lysate, an aliquot corresponding to 750 µl culture; **H**, eluate from amylose resin of ZAS-fusion protein, an aliquot corresponding to 750 µl culture; arrow indicates MBP-ZAS fusion protein. **(b)** UHPLC analysis of extracts obtained from incubation with crude lysate of MBP-ZAS producing cells, purified MBP-ZAS. Control assays were performed with crude lysate of MBP expressing cells. The incubation with apo-10'-zeaxanthinal (I) led to zaxinone (II). The UV-Vis spectra of substrate (I) and product (II) are shown in the insets, mAU, arbitrary units.

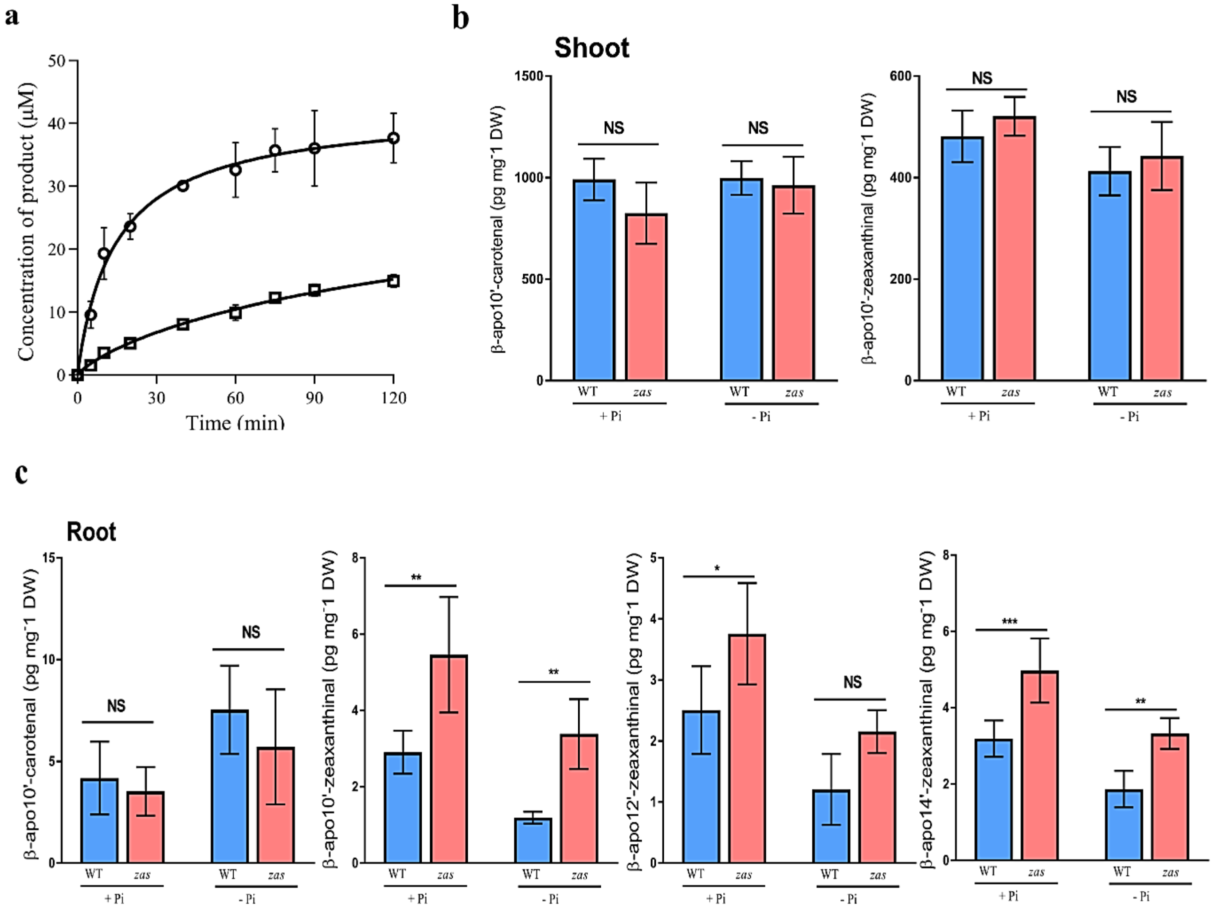

Supplementary Figure 3 | The specificity of apo-10'-zeaxanthinal for ZAS and profiling of apocarotenoids **(a)** *In vitro* the conversion rate curve of zaxinone from apo-10'-zeaxanthinal (*Circle*) and apo-8'-zeaxanthinal (*Square*). **(b)** Quantification of apo-10'-zeaxanthinal and β-apo10'-carotenal in WT and *zas* mutant shoots under normal (+Pi) and phosphorus deficient (-Pi) conditions **(c)** Profiling of β-apo10'-carotenal, apo-10'-, apo-12'- and apo-14'-zeaxanthinal in WT and *zas* mutant roots under normal and –Pi conditions. Data are the mean ± SD of n = 5 biological replicates. Statistical analysis was performed using One-way analysis of variance (ANOVA) and Tukey's *post hoc* test. Asterisk indicates significant differences, with a P value <0.05. ; NS, non-significant.

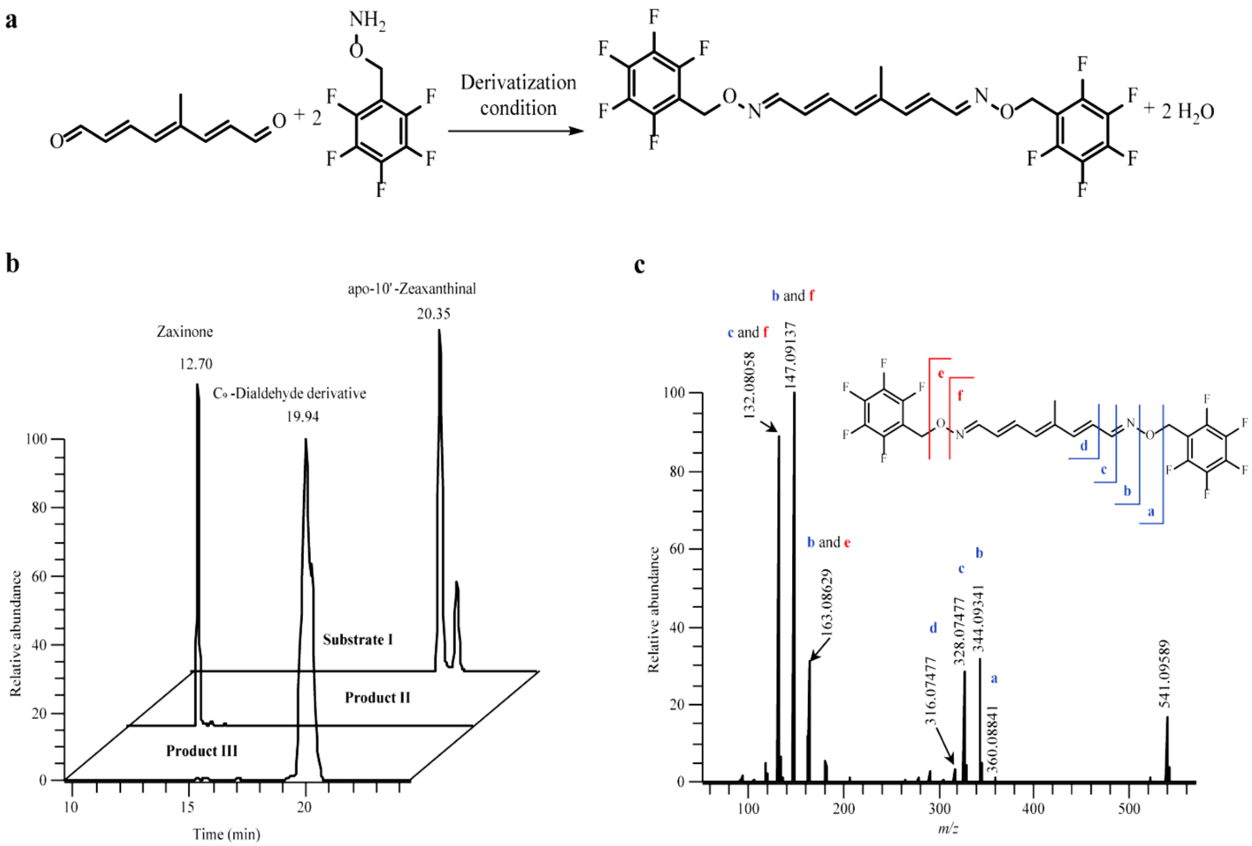

Supplementary Figure 4 | Identification of the C_9-dialdehyde produced from apo-10'-zeaxanthinal after derivatization reaction by using LC-MS/MS. **(a)** Derivatization reaction of C_9-dialdehyde**; (b)** The extracted ion chromatograms of apo-10'-Zeaxanthinal (I) zaxinone (II) and C_9-dialdehyde-derivative (III). (**c**) Full-scan spectrum of fragment ions of the C_9-dialdehyde-derivative is shown.

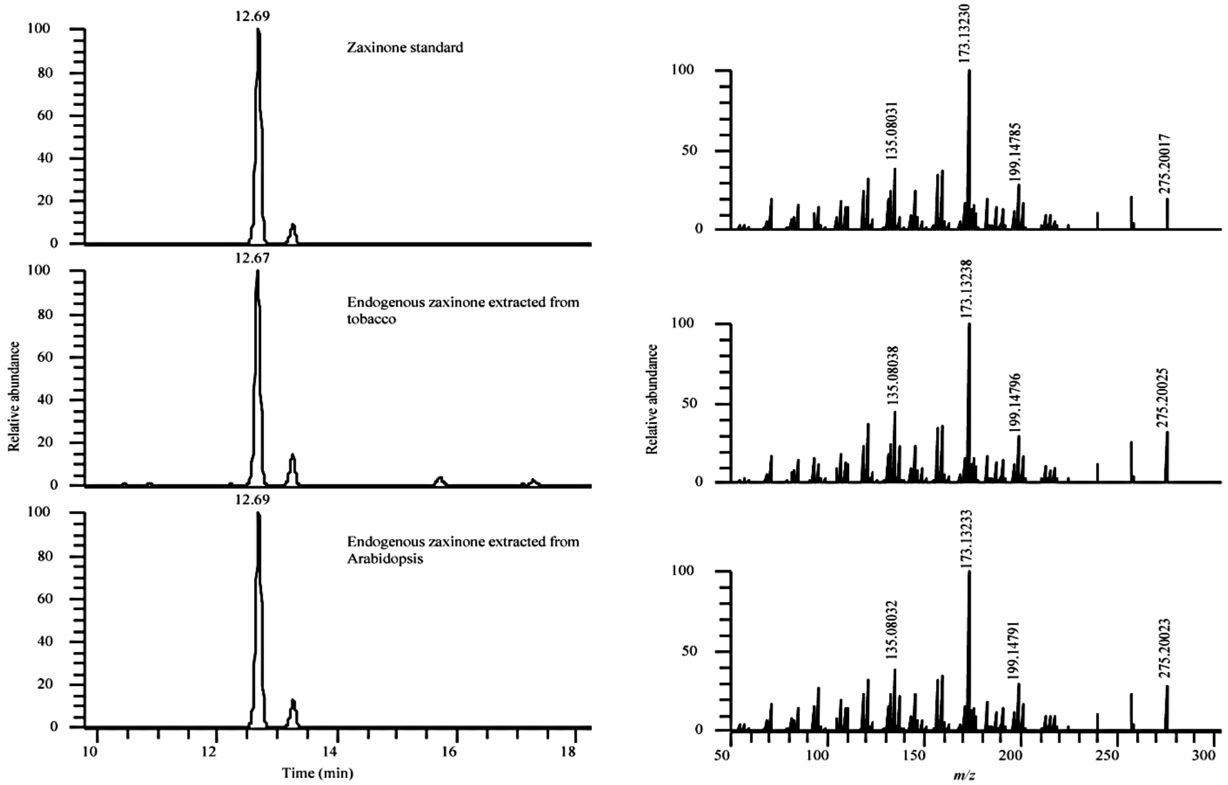

Supplementary Figure 5 | Identification of endogenous zaxinone from tobacco and Arabidopsis by using LC-MS/MS. Extracted ion chromatograms (*Left*) and full-scan spectra of fragment ions (*Right*) of zaxinone standard (*upper*), zaxinone extracted from tobacco (*middle*) and zaxinone extracted from Arabidopsis (*bottom*) are shown.

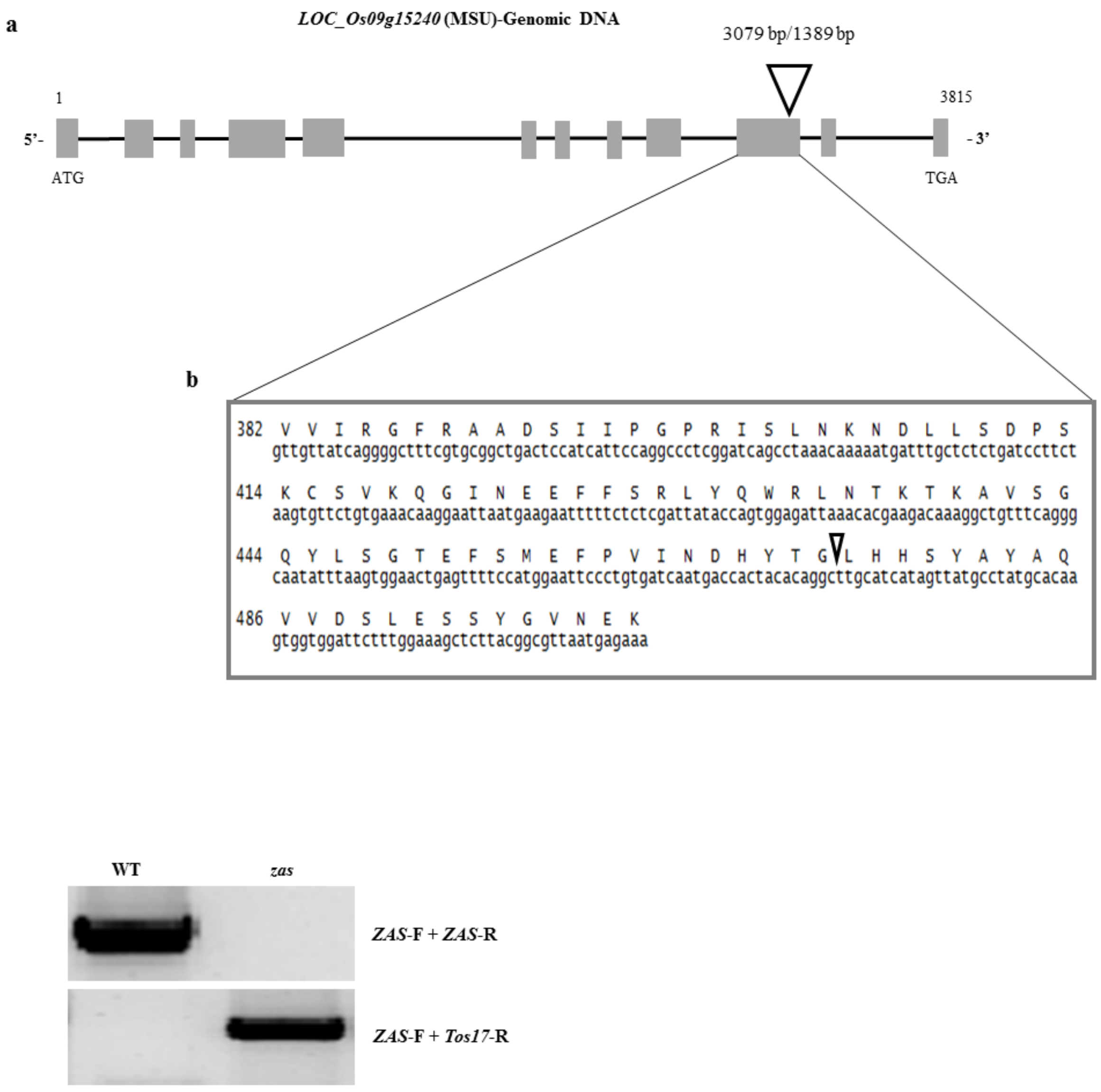

Supplementary Figure 6 | (a) Structure of the rice *ZAS* gene (*LOC*_Os09g15240-MSU) and the *Tos17* insertion site. Exons are indicated by the grey boxes separated by intron (solid lines). The A of ATG designates nucleotide 1 and the nucleotide triplet TGA indicates the stop codon. An arrow indicates the *Tos17* insertion site with its position in the genomic (3079 bp) and in the coding sequence (1389 bp). **(b)** Nucleotide and amino acid sequence of exon 10 carrying *Tos17* retrotranspon insertion is represented in the grey box. The arrow indicates the *Tos17* insertion site.**(c)** Homozygous *zas* rice mutant was verified by PCR assays using *ZAS*-specific primers (F+R) and *ZAS*- and *Tos17*-specific primers.

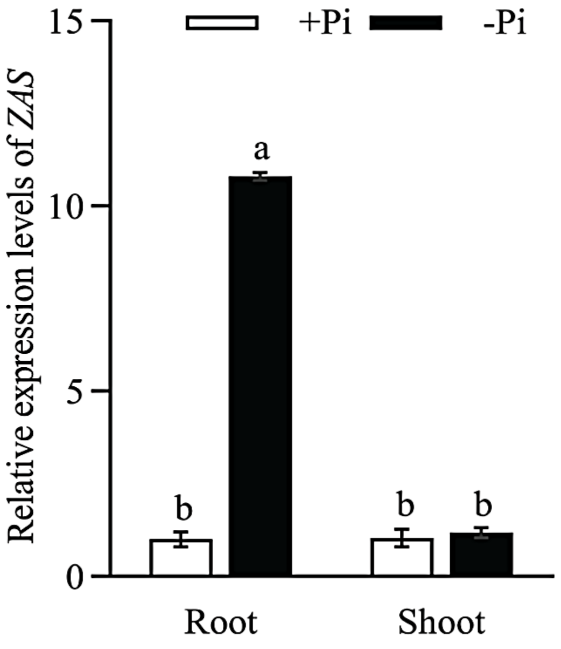

Supplementary Figure 7 | RT-qPCR analysis of the *Zaxinone Synthase (ZAS)* transcript levels. The relative expression levels of *ZAS* under normal (+Pi) and phosphate-starved (-Pi) conditions in Nipponbare wild-type (WT) root and shoot. The expression levels in the WT root were normalized to 1. Bars represent mean ± SD (n = 3 biological replicates). Statistical analysis was performed using One-way analysis of variance (ANOVA) and Tukey's *post hoc* test. Different letters denote significant differences ($P < 0.05$).

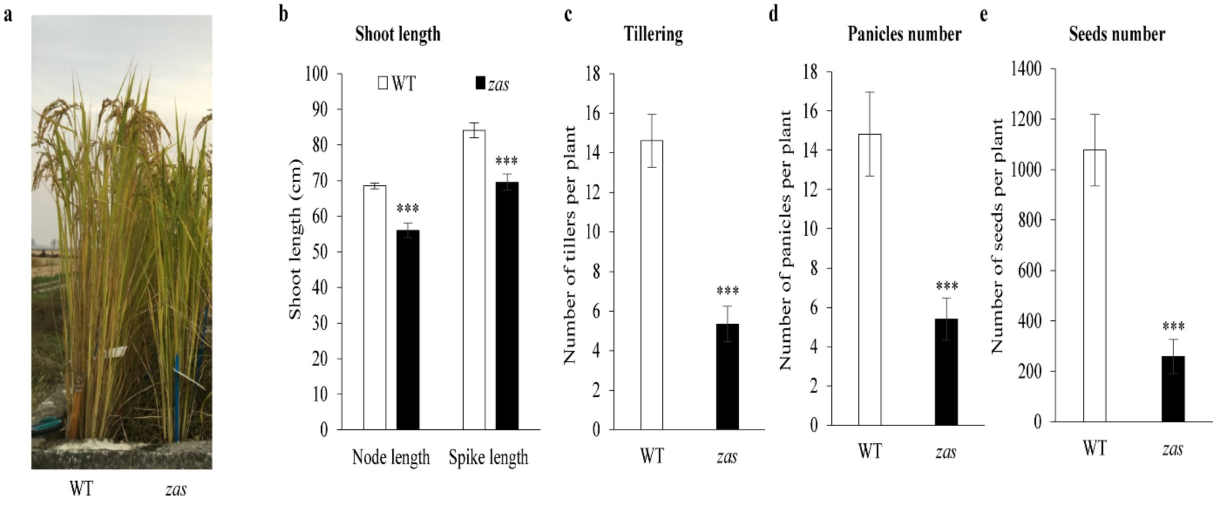

Supplementary Figure 8 | Above-ground phenotypic characterization of the *zas* mutant and wild-type grown in field conditions. Plants were grown in soil field and data were collected after five months (May-October). *zas* mutant showed a statistically significant reduction in all the morphological parameters analysed such as: shoot length **(b)**, in terms of node and spike length, tillering **(c)**, panicles **(d)** and seeds **(e)** number. Data are mean ± SE, $n = 12$ plants. Significant values (by *t*-test) are shown as follows: $***P < 0.001$.

a

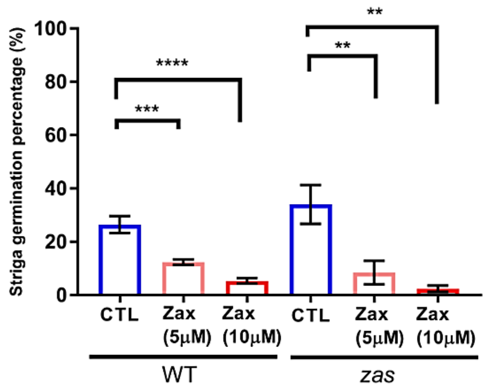

b

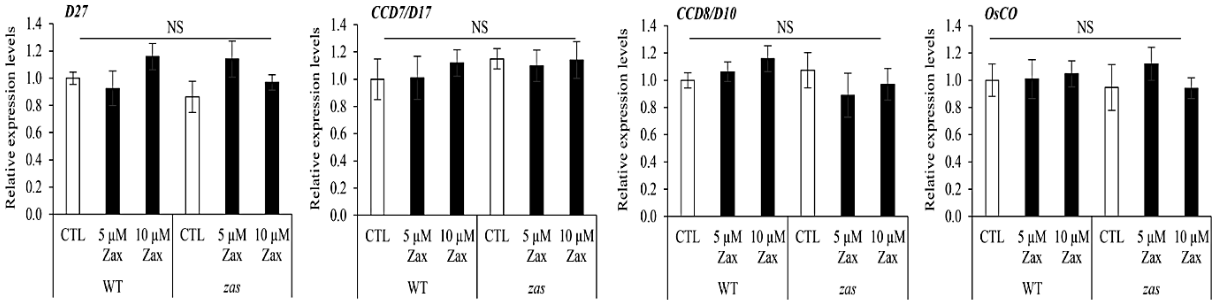

Supplementary Figure 9| Effect of zaxinone on *Striga* seed germinating activity of root exudates and transcript levels of SL biosynthetic genes of Nipponbare wild-type (WT) and *zas* mutant under normal (+Pi) conditions. **(a)** Percentage of *Striga hermonthica* seed germination upon application of 3-fold diluted root exudates. Data are mean ± SE, *n* = 5 (50 to 100 seeds per replicate).

(b) Transcript levels of SL biosynthesis genes (*D27, CCD7, CCD8* and *CO (Carlactone oxidase*)) in root tissues. The expression levels were detected by qRT-PCR. *Ubiquitin* was used as a reference gene, and the expression levels in the WT-control were normalized to 1. Bars represent mean ± SD (*n* = 3 biological replicates). Control treatment (0.1 % acetone). Statistical analysis was performed using One-way analysis of variance (ANOVA) and Tukey's *post hoc* test. Asterisk indicates significant differences, with a *P* value <0.05. ; NS, non-significant. CTL, Control; Zax, Zaxinone.

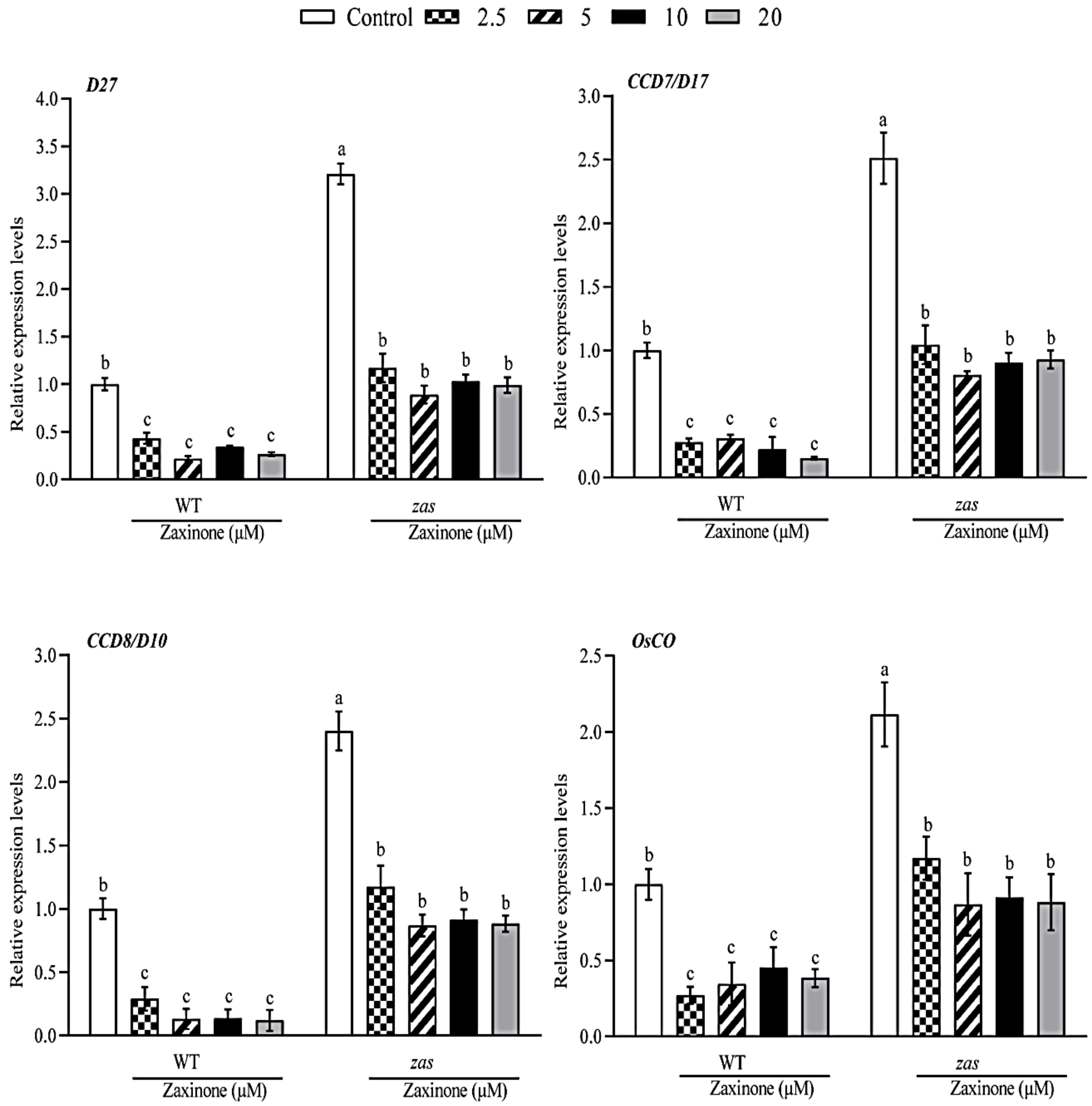

Supplementary Figure 10 | Effects of zaxinone on transcript levels of SL biosynthesis genes in Pi-starved (-Pi) rice roots. SL biosynthetic (*D27, CCD7, CCD8* and *CO* (*Carlactone oxidase*)) genes expression in WT and *zas* mutant. Control treatment (0.1 % acetone). The expression levels were detected by quantitative RT-PCR. *Ubiquitin* was used as a reference gene and transcript levels in the WT-control were normalized to 1. Bars represent mean ± SD (n = 3 biological replicates). Statistical analysis was performed using One-way analysis of variance (ANOVA) and Tukey's *post hoc* test. Different letters denote significant differences ($P < 0.05$).

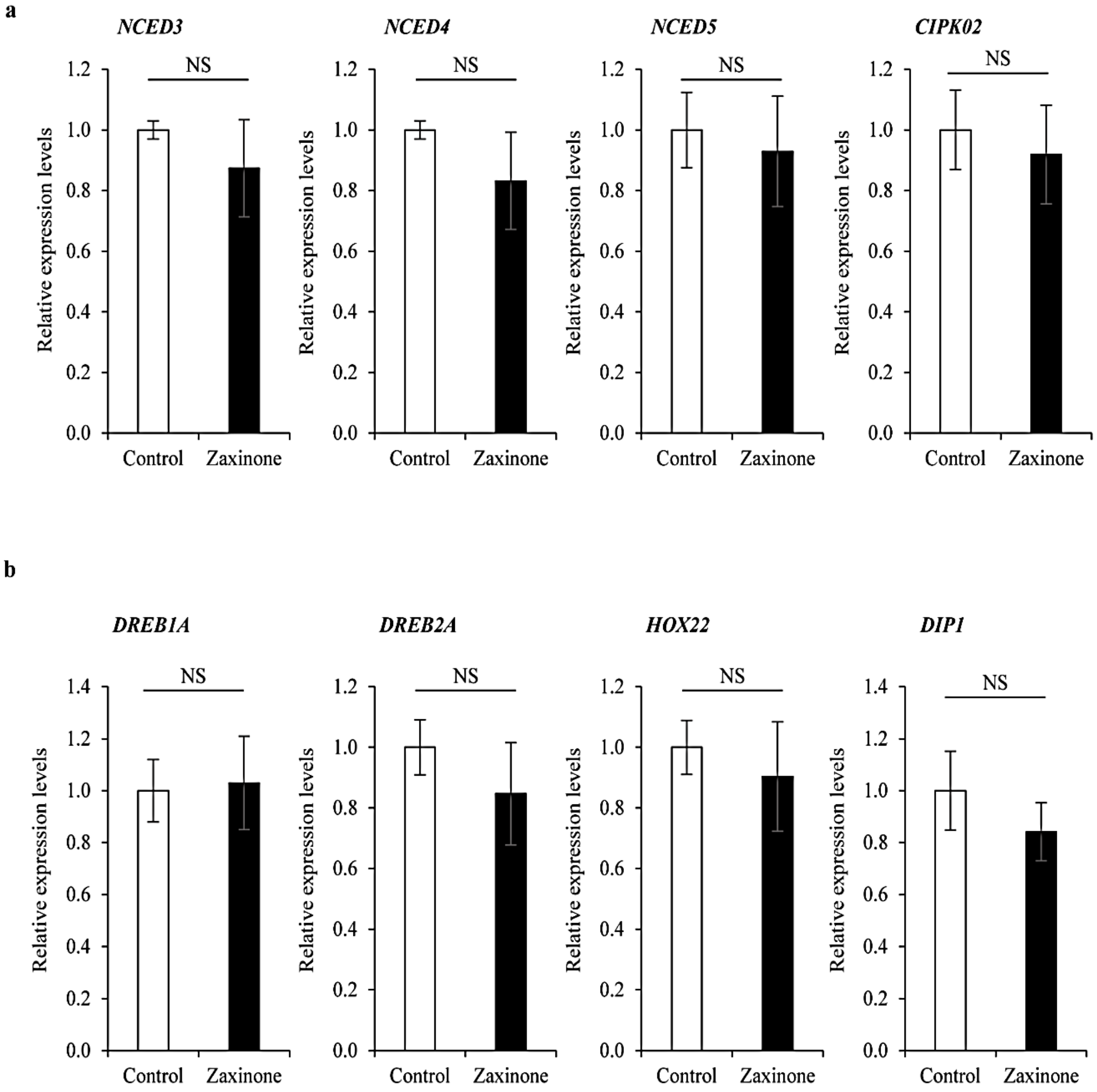

Supplementary Figure 11 | Effects of zaxinone (5 µM) on transcript levels of ABA and stress-responsive genes in roots of phosphate-starved (-Pi) Nipponbare rice seedlings.

(**a**) Transcript levels of the ABA biosynthesis key enzymes, 9-*cis*-epoxycarotenoid dioxygenase 3, 4 and 5 (NCED3, NCED4 and NCED5)[1] and of the ABA responsive protein, calcineurin B-like protein-interacting protein kinases (CIPK02)[2]. (**b**) Transcript levels of the abiotic stress-responsive genes, *dehydration responsive element binding protein 1A* and *2A* (*DREB1A* and *DREB2A*) [3], *HD-ZIP I class homeobox* (*HOX22*)[4], and dehydration stress-inducible protein (*DIP1*)[5]. Control treatment (0.1 % acetone). The expression levels were detected by qRT-PCR. Ubiquitin was used as a reference gene, and transcript levels in the control were normalized to 1. Bars represent mean ± SD (n = 3 biological replicates). Statistical analysis was performed using One-way analysis of variance (ANOVA) and Tukey's post hoc test. NS, non-significant. Rice MSU (http://rice.plantbiology.msu.edu/) locus IDs: *NCED3* (Os03g44380); *NCED4* (Os07g05940); *NCED5* (Os12g42280); *CIPK02* (Os07g48100); *DREB1A* (Os09g35030); *DREB2A* (Os01g07120); *HOX22* (Os04g45810); *DIP1* (Os02g44870).

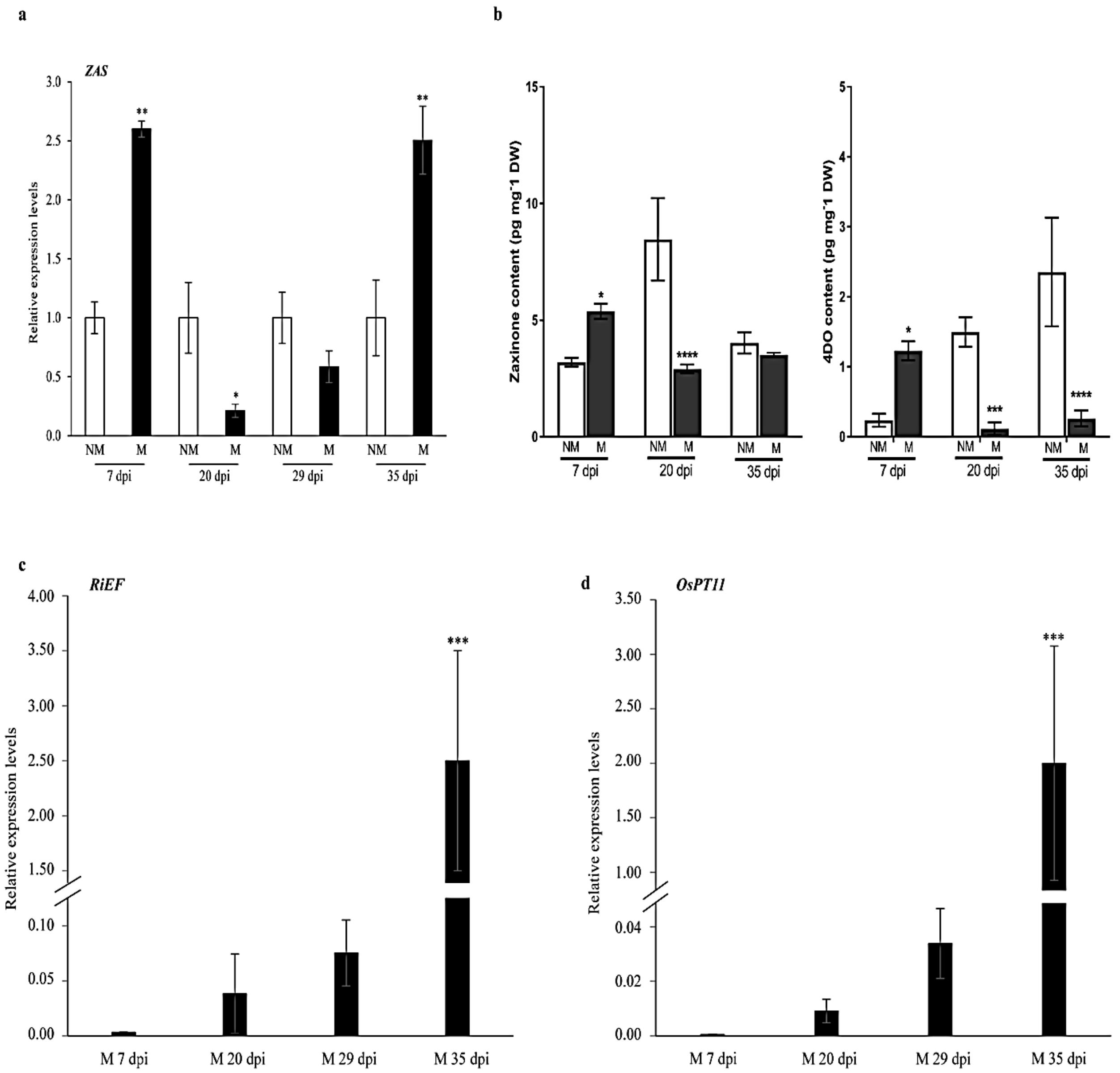

Supplementary Figure 12 | *ZAS* expression analysis assessed by qRT-PCR in a time course experiment of wild-type mycorrhizal root. **(a)** The relative expression of *ZAS* was evaluated in qRT-PCR during a time-course experiment performed on Nipponbare wild-type (WT) roots, colonized by *R. irregularis* from 7 to 35 days-post-inoculation (dpi). **(b)** Quantification of endogenous zaxinone and 4-deoxyorobanchol (4-DO) in wild-type roots colonized by *R. irregularis*, from 7 to 35 days-post-inoculation (dpi). **(c-d)** The relative expression of *RiEF* (fungal housekeeping) and *OsPT11* (AM marker gene) was evaluated in qRT-PCR to evaluate the level of mycorrhization in the different time points. [(a), n=4; (b), n=3; (c), n=4; (d), n=4]. Data are the average of four biological replicates and the bars represent ± SE.One-way analysis of variance (ANOVA) and Tukey's *post hoc* test were performed considering data coming from single time point. Asterisks indicate significant differences, *$P < 0.05$, **$P < 0.01$, ***$P < 0.001$, ****$P < 0.0001$. NM: non-mycorhizal roots; M: mycorrhizal roots.

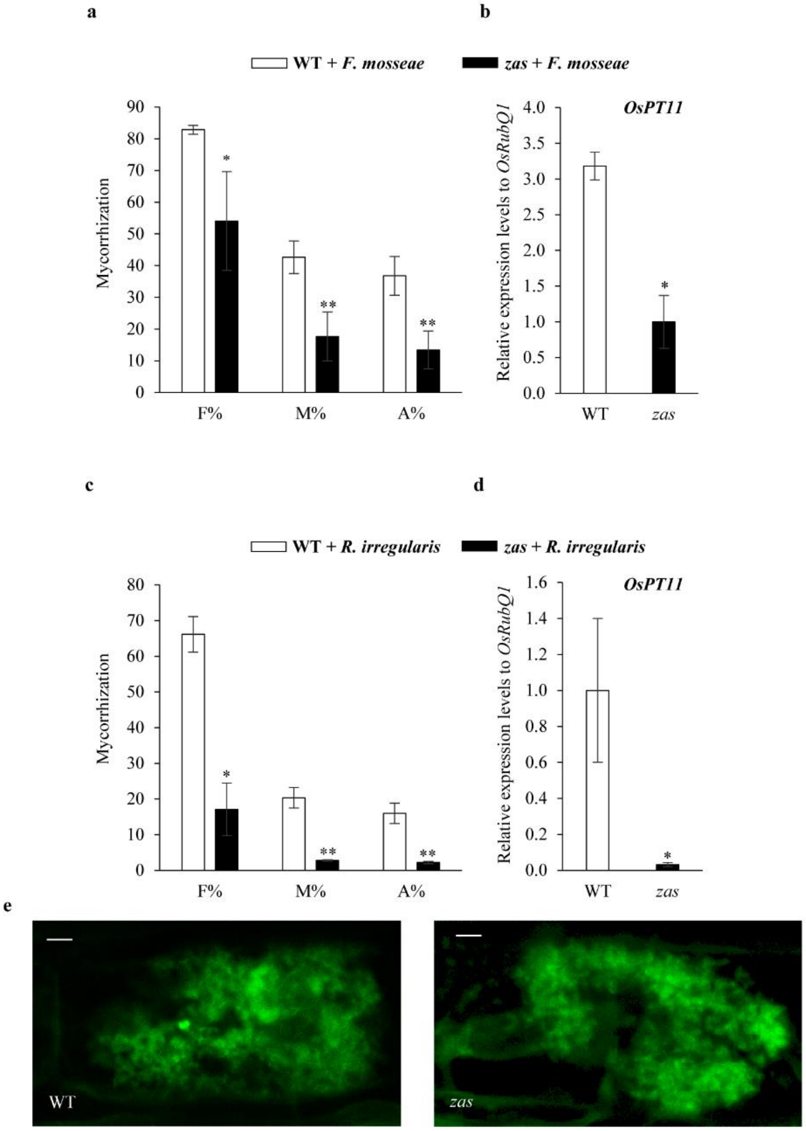

Supplementary Figure 13 | Mycorrhizal colonization in Nipponbare wild-type (WT) and *zas* mutant by the AM fungi *Funneliformis mosseae and Rhizophagus irregularis* at 35 dpi. **(a, c)** Degree of colonization expressed as mycorrhizal frequency (F %), intensity (M %) and arbuscule abundance (A %) in the root system of both WT and *zas* mutant plants. Data are the average of five biological replicates and ± SE are represented by bars. Asterisks indicate significant differences (One-way ANOVA test, $P < 0.05$). **(b, d)** Expression level of *OsPT11* gene, a plant marker of a functional symbiosis, analyzed by qRT-PCR in mycorrhizal roots of WT and *zas* mutant plants. Data are the average of four biological replicates and the bars represent ± SE. Asterisk indicates significant differences, with a P value <0.05. **(e)** Cortical cells from WT and *zas* mutant plants containing arbuscules, n=3 biological replicate samples containing different root sections were inspected. A total number of about 30 arbuscules were analyzed for each genotypes. No differences in arbuscules morphology were detected: green fluorescence is due to wheat germ agglutinin-fluorescein isothiocyanate. Scale bars: 10 µm.

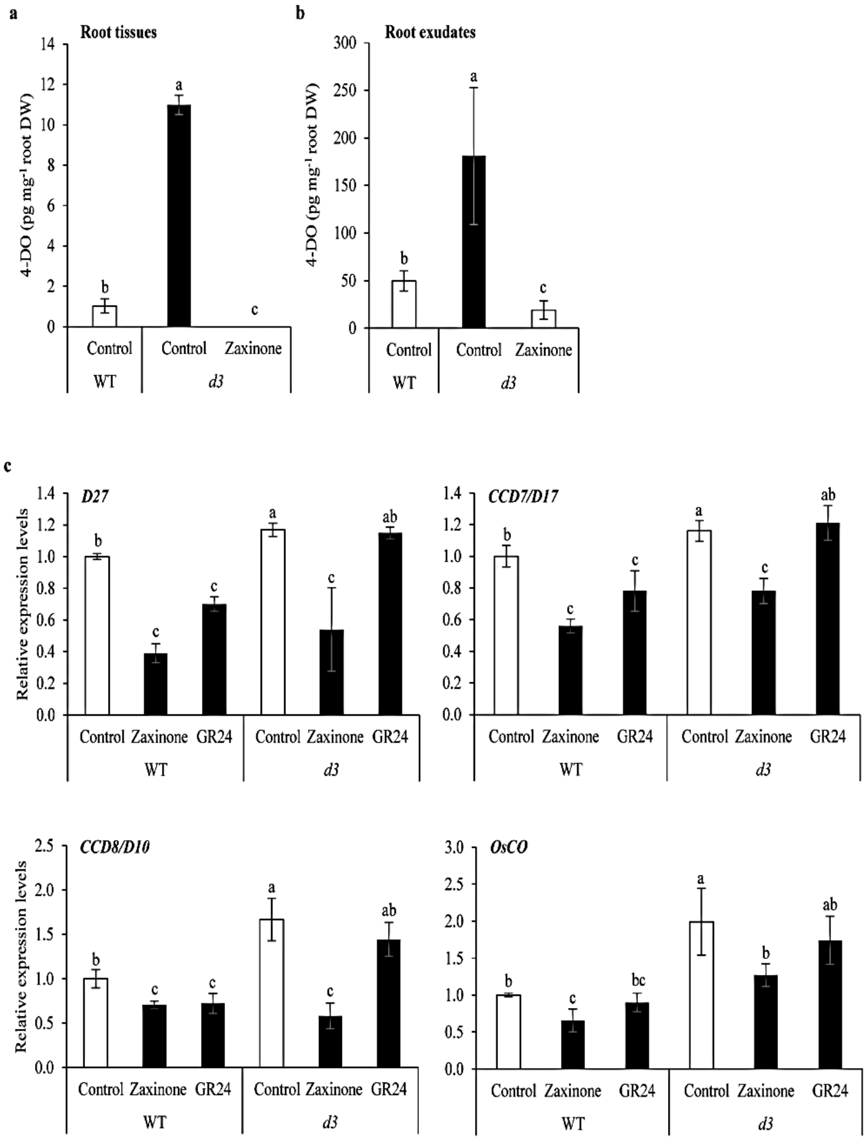

Supplementary Figure 14 | Effects of zaxinone on SL content and release, and transcript levels of SL biosynthesis genes in Pi-starved (-Pi) rice roots of Shiokari wild-type (WT) and *d3* mutant. (a-b) Amount of 4-DO (4-deoxyorobanchol) in root tissues **(a)** and root exudates **(b)** in response to 5 µM zaxinone treatment. **(c)** Transcript levels of SL biosynthesis genes (*D27, CCD7, CCD8* and *CO* (*Carlactone oxidase*) in root tissues collected for SL quantification. The expression levels were determined by qRT-PCR. *Ubiquitin* was used as a reference gene and the expression levels in the WT- control were normalized to 1. Two week-old seedlings were grown for another one week in – Pi solution, then transferred to new vials containing fresh – Pi solution with and without 5 µM of zaxinone and *rac*-GR24 for 6 h, then samples were collected. Bars represent mean ± SD (*n* = 3 biological replicates). Statistical analysis was performed using One-way analysis of variance (ANOVA) and Tukey's *post hoc* test. Different letters denote significant differences ($P < 0.05$).

Supplementary References

1. S. Hwang et al., heterologous expression of rice 9-cis-epoxycarotenoid dioxygenase 4 (OsNCED4) in arabidopsis confers sugar oversensitivity and drought tolerance. *Bot Stud*. **59:2** (2018)
2. Y. Xiang et al., Characterization of stress-responsive CIPK genes in rice for stress tolerance improvement. *Plant Physiol.* **144**, 1416-1428 (2007.)
3. J. Dubouzetet al., OsDREB genes in rice, Oryza sativa L., encode transcription activators that function in drought-, high-salt- and cold-responsive gene expression. *Plant Biotechnol. J.* **33**, 751-763 (2003)
4. S. Zhang et al., Function of the HD-Zip I gene Oshox22 in ABA-mediated drought and salt tolerances in rice. *Plant Mol Biol*. **6**, 571-585 (2012)
5. I. Jang et al., Expression of a bifunctional fusion of the *Escherichia coli* genes for trehalose-6-phosphate synthase and trehalose-6-phosphate phosphatase in transgenic rice plants increases trehalose accumulation and abiotic stress tolerance without stunting growth. *Plant Physiol.* **131**, 516-524. (2003)
