# Supplementary material for: The apocarotenoid metabolite zaxinone regulates growth and strigolactone biosynthesis in rice
Source: Nat Commun. 2019 Feb 18;10:810. doi: 10.1038/s41467-019-08461-1 (PMC6379432; doi:10.1038/s41467-019-08461-1)
Supplement: Supplementary file 2 — Description of Additional Supplementary Files [file 41467_2019_8461_MOESM2_ESM.pdf]

Description of Additional Supplementary Files

File Name: Supplementary Data 1
Description: Distribution of CCD orthologues across land plants. Abreviation: SN, shorten name of the species used in phlygenetic trees of this study. Columns from 5th to 10th represent numbers of ZAS, NCED, CCD1, CCD4, CCD7 and CCD8 orthologues in each species' genome.

File Name: Supplementary Data 2
Description: Distribution of sub groups of ZAS orthologues across land plants. Abreviation: SN, shorten name of the species used in phlygenetic trees of this study. Columns from 5th to 14th represent numbers of Group1-Group10 orthologues in each species' genome.

File Name: Supplementary Data 3
Description: Distribution of CCD orthologues of non-AM host plants. Abreviation: SN, shorten name of the species used in phlygenetic trees of Supplementary Data 6. Columns from 5th to 10th represent numbers of NCED, CCD1, CCD4, CCD7, CCD8 and ZAS orthologues in each species' genome.

File Name: Supplementary Data 4
Description: Primer sequences used in this study

File Name: Supplementary Data 5
Description: CCD Sequences of Spirodela polyrhiza and Utlicularia gibba

File Name: Supplementary Data 6
Description: Neighbour-joining tree of plant CCD orthologues (dendrogram). Sub clusters (a) the whole tree (schematic), (b) NCED, (c) CCD1, (d) CCD4, (e) ZAS, (f) CCD7 and (g) CCD8 formed in the tree of Fig. 1B. Species names corresponding to shorten names in the tree are described in the Supplementary Data 2. The scale bar on the bottom right of each figure indicates estimated substitutions per amino acid. Bootstrap values more than 50 % are only shown in the tree.

File Name: Supplementary Data 7
Description: (a) Neighbour-joining tree of ZAS orthologues re-constructed using 106 sequences belonging to ZAS sub cluster in Fig. 1b. Sequence names are composed of two parts separated by a space. The former one represents shorten names of species whereas the latter one describes NCBI protein sequence ID. Species names corresponding to shorten names in the tree are described in Supplementary Data 1. The scale bar indicates estimated 0.1 change per amino acid. Bootstrap values more than 50 % are only shown in the tree. (b) Neighbour-joining tree of A. thaliana and O. sativa CCD orthologues. At and Os in sequence names represent A. thaliana and O. sativa respectively and XP or NP numbers represent protein sequence IDs in NCBI. The scale bar indicates estimated 0.1 change per amino acid. Bootstrap values more than 50 % are only shown in the tree.

File Name: Supplementary Data 8
Description: Neighbour-joining tree of CCDs in no-AM host plants. Red branches represent sequences of non-AM host plants, whereas black branches indicate AM host plant sequences. Sequence names are composed of two parts separated by a space. The former one represents shorten names of species whereas the latter one describes protein sequence ID in each database. CCD sequences of U. gibba and S. polyrhiza predicted in this study were described in Supplementary Data 5. Species names corresponding to shorten names in the tree are described in Supplementary Data 3. Bootstrap values more than 50% were only shown.
