# Supplementary figures and images for: The apocarotenoid metabolite zaxinone regulates growth and strigolactone biosynthesis in rice
Source: Nat Commun. 2019 Feb 18;10:810. doi: 10.1038/s41467-019-08461-1 (PMC6379432; doi:10.1038/s41467-019-08461-1)

**a**

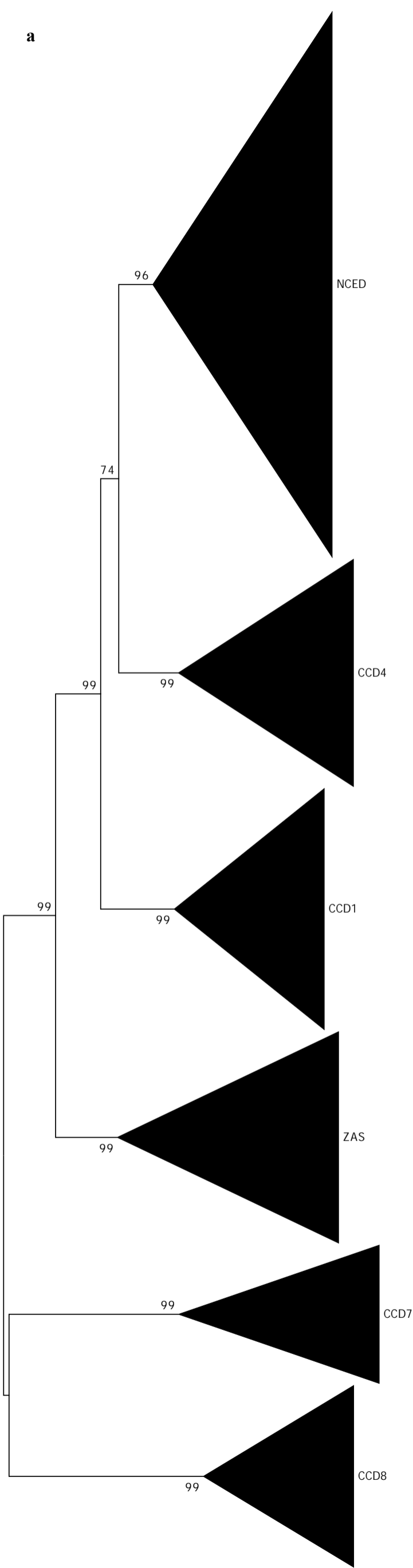

0.1

**b**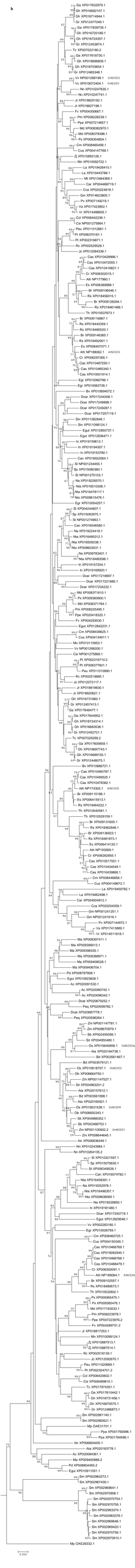

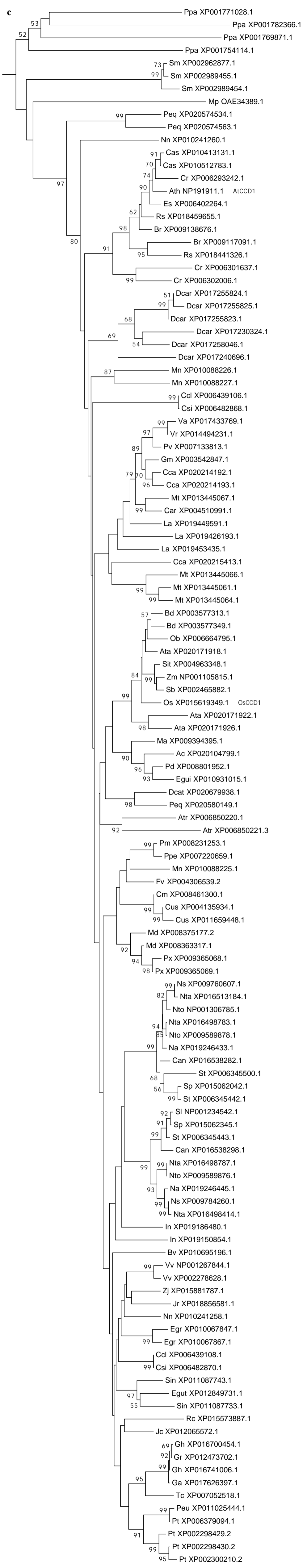

0.050

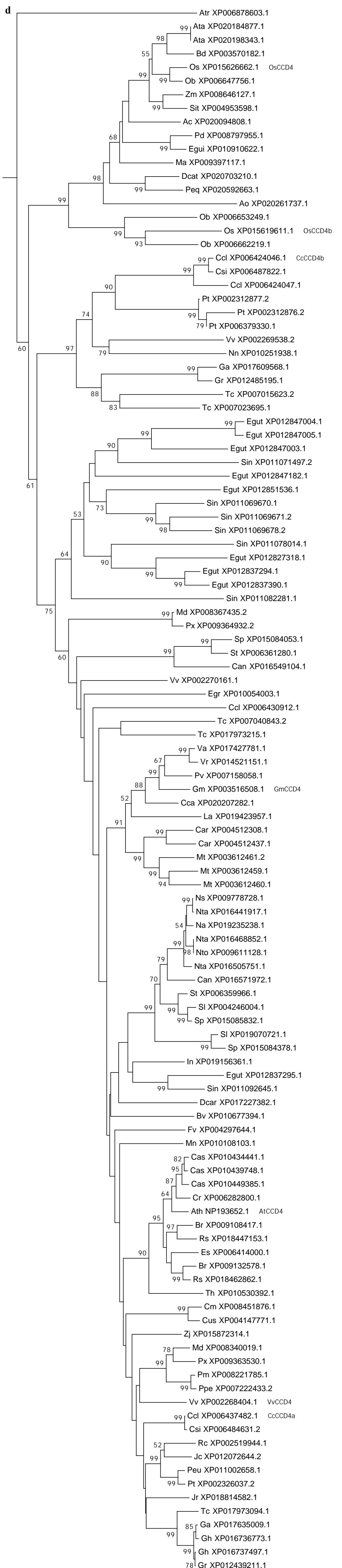

0.050

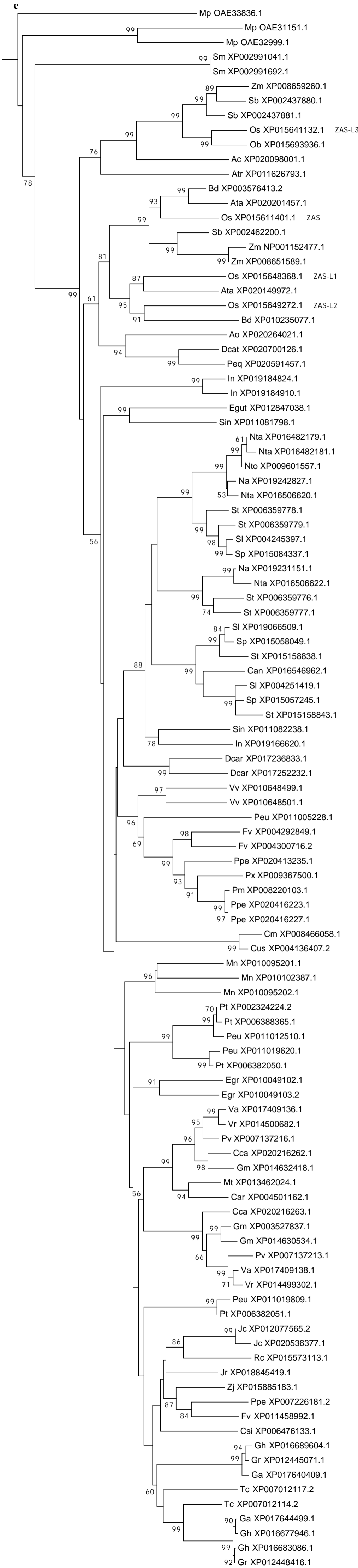

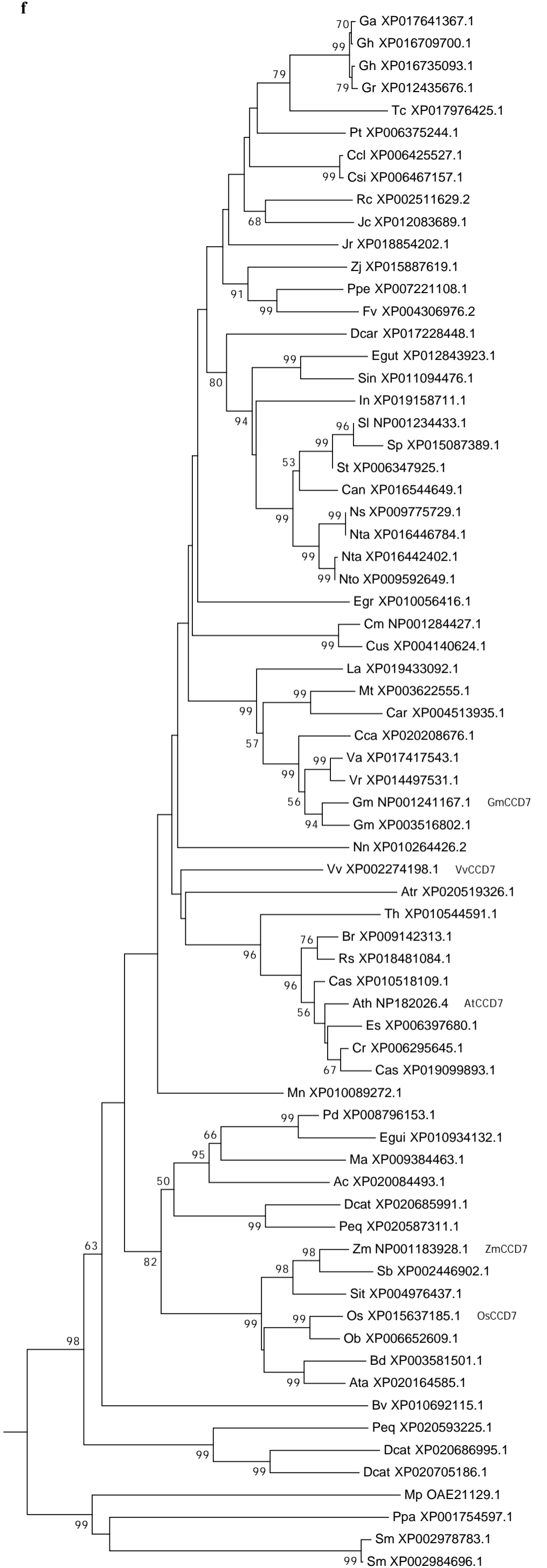

0.10

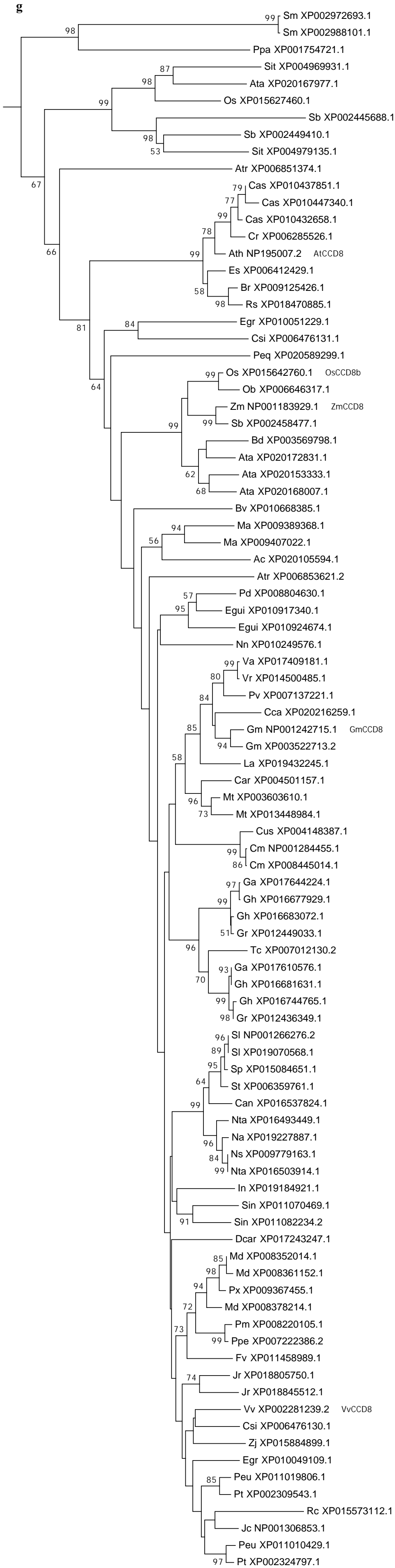

0.050

Supplement: Supplementary file 8 — Supplementary Data 6 [file 41467_2019_8461_MOESM8_ESM.pdf]

**a**

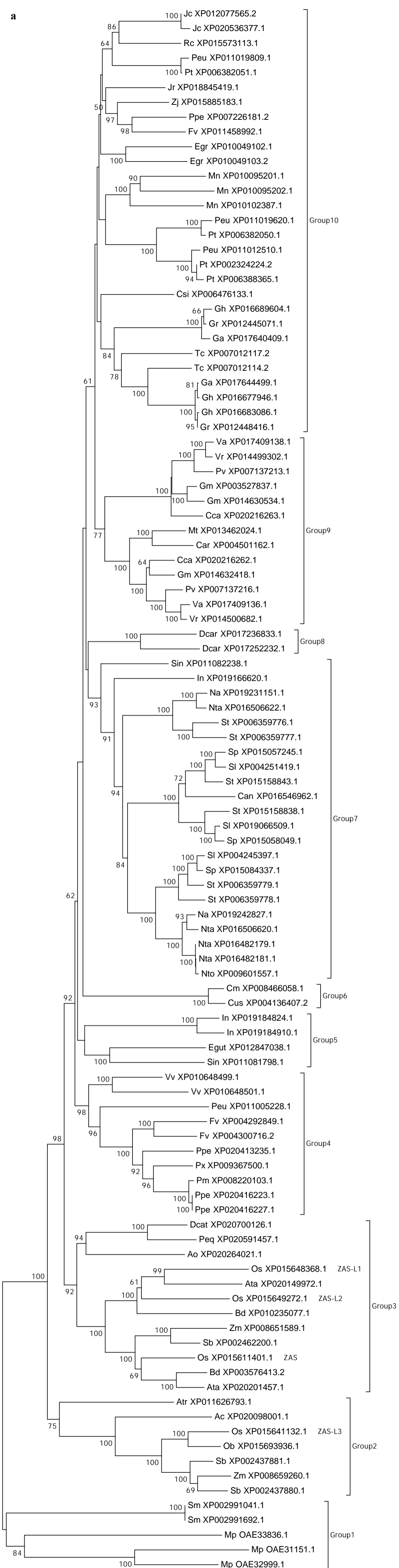

**b**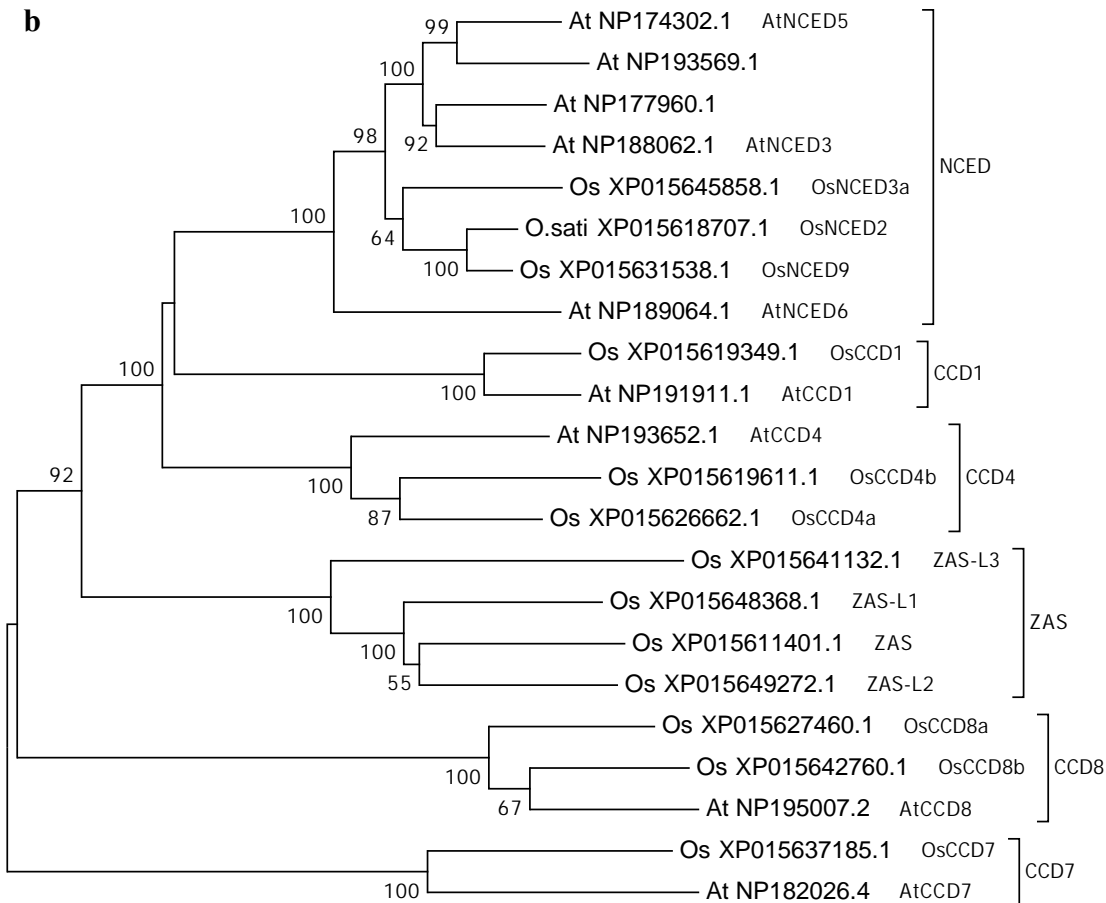

0.1

Supplement: Supplementary file 9 — Supplementary Data 7 [file 41467_2019_8461_MOESM9_ESM.pdf]

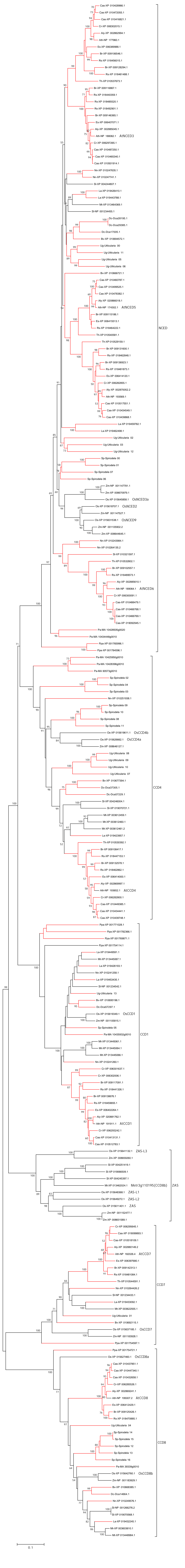

Supplement: Supplementary file 10 — Supplementary Data 8 [file 41467_2019_8461_MOESM10_ESM.pdf]

## Slide 1
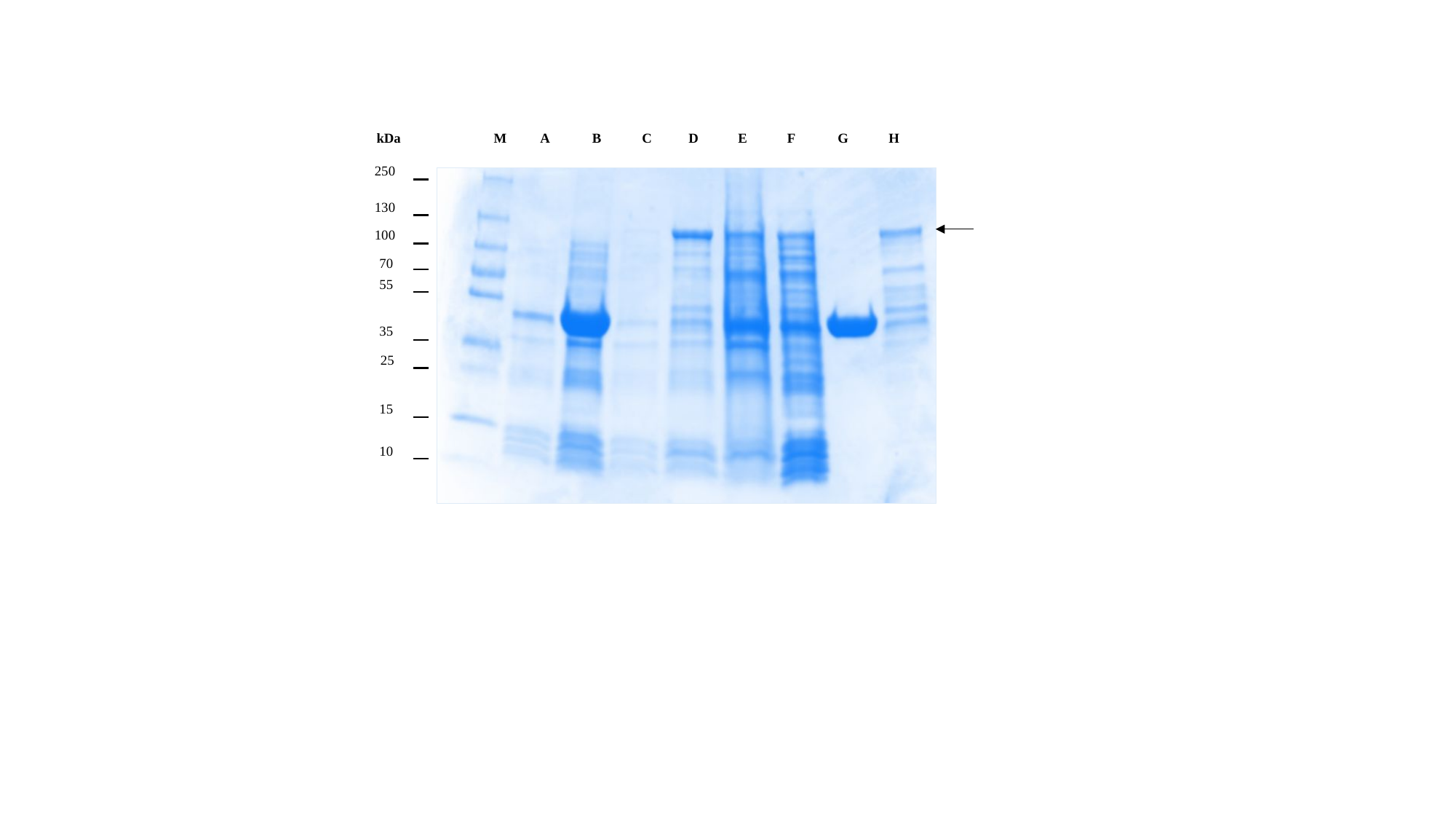

kDa
M
A
B
C
D
E
F
G
H
250
130
100
70
55
35
25
15
10

Supplement: Supplementary file 11 — Source Data [file 41467_2019_8461_MOESM11_ESM.zip › Fig. S2_Purified ZAS protein gel.pptx]
