# Supplementary material for: The apocarotenoid metabolite zaxinone regulates growth and strigolactone biosynthesis in rice
Source: Nat Commun. 2019 Feb 18;10:810. doi: 10.1038/s41467-019-08461-1 (PMC6379432; doi:10.1038/s41467-019-08461-1)
Supplement: Supplementary file 11 — Source Data [file 41467_2019_8461_MOESM11_ESM.zip › Fig. S6C_Screening homozygous zas mutant_PCR.pptx]

## Slide 1
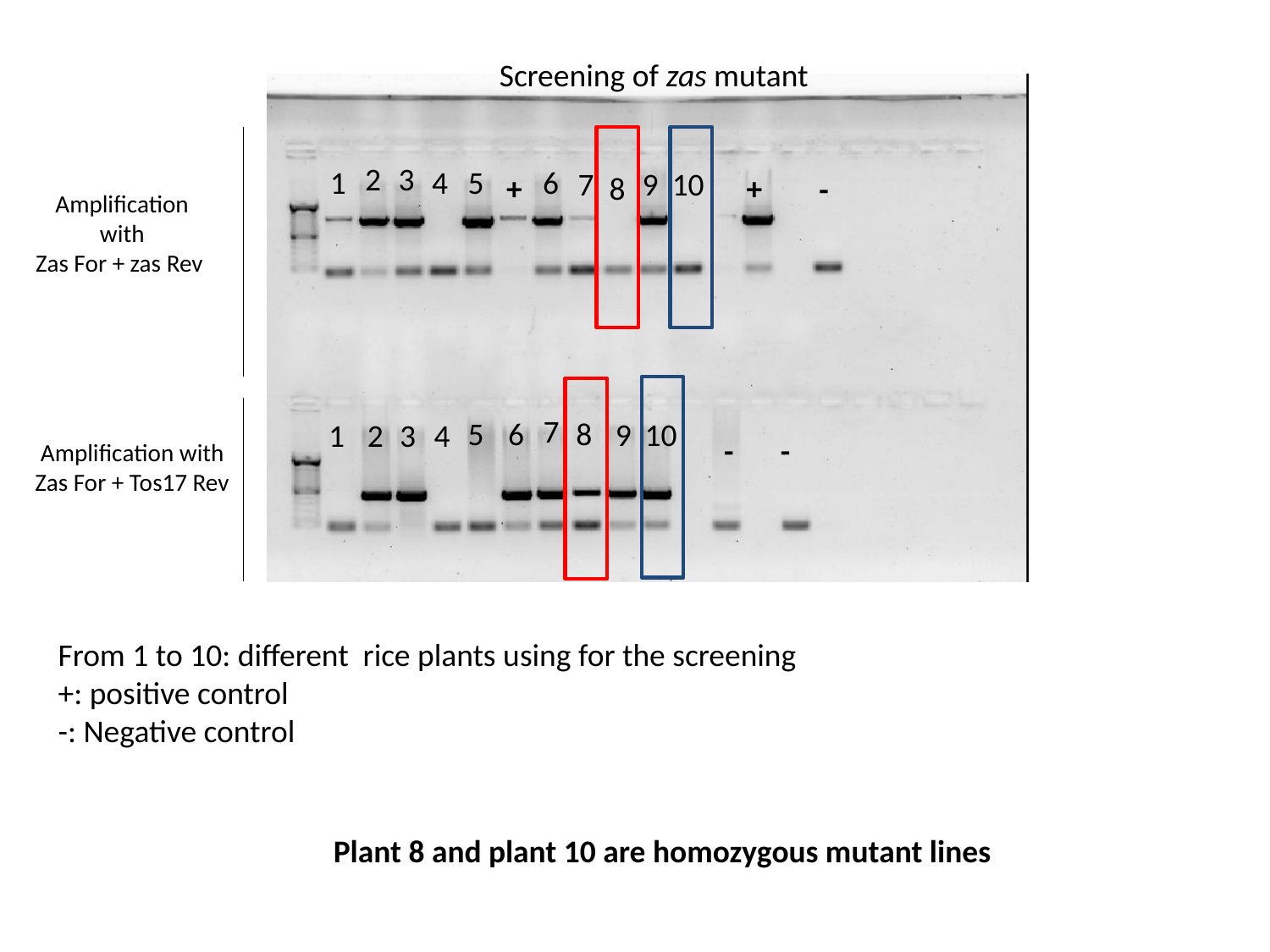

Screening of zas mutant
3
2
1
4
5
6
10
7
9
+
8
+
-
Amplification with
Zas For + zas Rev
7
5
8
6
10
9
1
2
3
4
-
-
Amplification with
Zas For + Tos17 Rev
From 1 to 10: different rice plants using for the screening
+: positive control
-: Negative control
Plant 8 and plant 10 are homozygous mutant lines
