# Supplementary material for: The apocarotenoid metabolite zaxinone regulates growth and strigolactone biosynthesis in rice
Source: Nat Commun. 2019 Feb 18;10:810. doi: 10.1038/s41467-019-08461-1 (PMC6379432; doi:10.1038/s41467-019-08461-1)
Supplement: Supplementary file 12 — Reporting Summary [file 41467_2019_8461_MOESM12_ESM.pdf]

nature research

Corresponding author(s): NCOMMS-18-20519

Reporting Summary

Nature Research wishes to improve the reproducibility of the work that we publish. This form provides structure for consistency and transparency in reporting. For further information on Nature Research policies, see Authors & Referees and the Editorial Policy Checklist.

Statistical parameters

When statistical analyses are reported, confirm that the following items are present in the relevant location (e.g. figure legend, table legend, main text, or Methods section).

n/a	Confirmed	
☐	☒	The exact sample size (*n*) for each experimental group/condition, given as a discrete number and unit of measurement
☐	☒	An indication of whether measurements were taken from distinct samples or whether the same sample was measured repeatedly
☐	☒	The statistical test(s) used AND whether they are one- or two-sided<br>*Only common tests should be described solely by name; describe more complex techniques in the Methods section.*
☒	☐	A description of all covariates tested
☐	☒	A description of any assumptions or corrections, such as tests of normality and adjustment for multiple comparisons
☐	☒	A full description of the statistics including central tendency (e.g. means) or other basic estimates (e.g. regression coefficient) AND variation (e.g. standard deviation) or associated estimates of uncertainty (e.g. confidence intervals)
☐	☒	For null hypothesis testing, the test statistic (e.g. *F*, *t*, *r*) with confidence intervals, effect sizes, degrees of freedom and *P* value noted<br>*Give P values as exact values whenever suitable.*
☒	☐	For Bayesian analysis, information on the choice of priors and Markov chain Monte Carlo settings
☒	☐	For hierarchical and complex designs, identification of the appropriate level for tests and full reporting of outcomes
☒	☐	Estimates of effect sizes (e.g. Cohen's *d*, Pearson's *r*), indicating how they were calculated
☐	☒	Clearly defined error bars<br>*State explicitly what error bars represent (e.g. SD, SE, CI)*

Our web collection on statistics for biologists may be useful.

Software and code

Policy information about availability of computer code

Data collection	Protein fasta files of land plant genomes were obtained from NCBI RefSeq assembly (GCF_ prefixes) and NCBI Genbank assembly (GCA_ prefix). Protein fasta files of Picea abies were obtained from ConGenIE (Conifer Genome Integrative Explorer) web resource (http://congenie.org). Protein fasta files of Dianthus caryophyllus was retrieved from canation.kazusa.or.jp. Genome fasta files (Scaffold or contig levels) of Utricularia gibba (GCA_002189035.1), Spirodela polyrhiza (GCA_001981405.1) were retrieved from GenBank database. Obtained or predicted protein sequences were annotated by Pfam-A data base using Hmmscan program included in HMMER v3.1b1 package (http://www.hmmer.org/).
Data analysis	Software R, PAST statistical package version 2.16, MAFFT ver. 3 with default settings, MYCOCALC (http://www2.dijon.inra.fr/mychintec/Mycocalc-prg/download.html).

For manuscripts utilizing custom algorithms or software that are central to the research but not yet described in published literature, software must be made available to editors/reviewers upon request. We strongly encourage code deposition in a community repository (e.g. GitHub). See the Nature Research guidelines for submitting code & software for further information.

Data

Policy information about availability of data

All manuscripts must include a data availability statement. This statement should provide the following information, where applicable:

- Accession codes, unique identifiers, or web links for publicly available datasets
- A list of figures that have associated raw data
- A description of any restrictions on data availability

All data generated or analyzed during this study are included in this article and its supplementary information files.

Field-specific reporting

Please select the best fit for your research. If you are not sure, read the appropriate sections before making your selection.

☒ Life sciences ☐ Behavioural & social sciences ☐ Ecological, evolutionary & environmental sciences

For a reference copy of the document with all sections, see nature.com/authors/policies/ReportingSummary-flat.pdf

Life sciences study design

All studies must disclose on these points even when the disclosure is negative.

Sample size	Depending on the experiment, a minimum of 3 independent biological replicates was used to perform statistical analyses.
Data exclusions	No data were excluded.
Replication	All data were reproducible. At least three biological replicates were used in each experiment.
Randomization	Plants were randomized in growth chamber or greenhouse, and sample order was randomized when applying the compound.
Blinding	Blinding is not necessary because results are not subjective. Mutant phenotype and effects of compound treatment were quite obvious. Blinding was not possible in strigolactone quantification, as samples were analyzed immediately following treatments.

Reporting for specific materials, systems and methods

Materials & experimental systems

n/a	Involved in the study	
☒	☐	Unique biological materials
☒	☐	Antibodies
☒	☐	Eukaryotic cell lines
☒	☐	Palaeontology
☒	☐	Animals and other organisms
☒	☐	Human research participants

Methods

n/a	Involved in the study	
☒	☐	ChIP-seq
☒	☐	Flow cytometry
☒	☐	MRI-based neuroimaging
